# Lipid and Secondary Metabolite Profile Changes During Cold Storage of Dongzao (*Ziziphus jujuba* Mill. cv. Dongzao): Responses to Postharvest Treatments and Implications for Marketability

**DOI:** 10.3390/plants15213370

**Published:** 2026-11-01

**Authors:** Shengrui Yao, F. Omar Holguin

**Affiliations:** 1 Department of Plant and Environmental Sciences, New Mexico State University, Las Cruces, NM 88003, USA; frholgui@nmsu.edu; 2 Sustainable Agriculture Science Center at Alcalde, New Mexico State University, Alcalde, NM 87511, USA

**Keywords:** fungicides, Scholar, Mentor, physiological changes, lipids, secondary metabolism, flavonoids, terpenoids

## Abstract

Dongzao (*Ziziphus jujuba* Mill. cv. Dongzao) is a popular fresh-eating cultivar with a short shelf life at room temperature. As Dongzao acreage increases in the United States, extending the fresh-fruit supply window is important for commercial production. A preliminary study showed that Dongzao fruit began developing mold after approximately 5 weeks of cold storage. Therefore, a subsequent study evaluated the effects of an ethylene absorbent and two fungicides, Scholar and Mentor, on fruit quality, decay, and metabolomic changes during extended cold storage at 0 ± 0.5 °C. Compared with untreated fruit, the ethylene absorbent did not extend storage life or reduce decay, with more than 30% stem-end rot observed by week 6, suggesting that ethylene removal had limited effects on Dongzao storage deterioration. Both fungicides substantially reduced decay, but Scholar was more effective, with no stem-end rot detected after 9 weeks of storage. Heatmap and principal component analyses further showed that Scholar-treated fruit after 9 weeks clustered with fruit stored for 6 weeks, suggesting that Scholar treatment was associated with delayed metabolic changes accompanying advanced storage deterioration. Cold storage induced extensive lipid remodeling, with substantial increases in lipid metabolites, particularly lysophosphatidylcholines, lysophosphatidylethanolamines, and free fatty acids, indicating pronounced changes in membrane lipid metabolism under prolonged low-temperature storage. Cold storage also broadly altered secondary metabolism, including flavonoids, phenolic acids, alkaloids, and terpenoids. Although cold storage was the primary driver of metabolomic changes, Scholar treatment was associated with distinct alterations in flavonoid and terpenoid profiles after 9 weeks of storage. Together, the results demonstrate that Scholar treatment can substantially extend the cold-storage life of Dongzao fruit and suggest that delayed decay and senescence are accompanied by distinct metabolic changes during prolonged storage. To our knowledge, this is the first study of Dongzao cold storage conducted in the United States and the first to characterize untargeted metabolomic changes in Dongzao during extended cold storage, providing a foundation for future research on Dongzao cold storage.

## 1. Introduction

Jujube cultivar Dongzao (*Ziziphus jujuba* Mill. cv. Dongzao) is a late-maturing cultivar known for its crisp texture, juicy flesh, sweet flavor, and excellent fresh-eating quality [1]. The fruit resembles a small apple in shape and is therefore commonly referred to as Pingguozao (Apple Jujube) in China. Dongzao originated in the Bohai Bay region, particularly in Cangzhou, Hebei Province, and Binzhou, Shandong Province. Over time, it has been widely adopted in major jujube-producing regions, including Hebei, Henan, Shandong, Shanxi, Shaanxi, and Xinjiang [1]. Currently, Dongzao remains the dominant fresh-eating jujube cultivar in China and is also one of the most popular fresh-eating cultivars in the New Mexico State University (NMSU) jujube collection [2].

Fresh jujube fruits are less perishable than berries such as raspberries and blackberries, but they are not as suitable for long-term storage as apples; some apple cultivars can be stored for many months under controlled conditions. Fresh jujube fruit has a limited shelf life at room temperature and can typically be stored for 2–3 weeks under refrigeration. In contrast, dried jujube fruit can be stored for approximately one year at room temperature and for two years or longer under cold-storage conditions [3].

As more growers adopt jujube as a specialty fruit crop, the acreage of Dongzao is expected to increase in the United States. To support industry growth, additional marketing opportunities and an extended marketing season are needed. Postharvest cold storage could provide an effective means of extending fruit availability beyond the harvest period, thereby improving market access and profitability for growers.

Several studies in China have investigated jujube cold storage, focusing on the effects of cultivar, fruit maturity stage, storage temperature, and postharvest treatments on fruit quality, storage life, and physiological and biochemical changes. Various postharvest treatments, including nitric oxide (NO), 1-methylcyclopropene (1-MCP), and cold atmospheric plasma, have been evaluated for their potential to delay senescence, maintain fruit quality, and extend storage life [4,5,6,7,8,9,10]. However, most of these studies were conducted under Chinese production and storage conditions, and information on the postharvest behavior and cold-storage potential of ‘Dongzao’ under U.S. growing and storage conditions remains limited. Kader et al. (1982) investigated the storage behavior of an unspecified jujube cultivar held at 20 °C for 15 days and reported that jujube exhibits a non-climacteric ripening pattern, with no climacteric increase in ethylene production during ripening [11]. At New Mexico State University (NMSU), preliminary cold-storage studies conducted in 2014 with cultivars ‘Li’ and ‘Sherwood’ at 4 °C indicated that ‘Sherwood’ fruit could be stored longer than ‘Li’ fruit [12].

Mass spectrometry (MS)-based metabolomics is a powerful approach for the comprehensive profiling of metabolites in biological samples [13]. Recent metabolomic studies have characterized a broad spectrum of metabolites in jujube fruit and seeds, providing a more comprehensive understanding of their metabolic composition [14,15]. In contrast, most previous jujube cold-storage studies have focused on changes in individual quality and nutritional attributes, such as sugars, vitamin C, antioxidants, and titratable acidity. Comprehensive monitoring of metabolite changes during cold storage could provide additional insights into the physiological responses of jujube fruit to prolonged storage and may help identify metabolic changes associated with fruit senescence, quality deterioration and storage life.

Given the commercial importance of ‘Dongzao’ and the lack of storage information under U.S. growing conditions, a cold-storage study was conducted to evaluate changes in fruit quality and storage potential during extended cold storage. In a preliminary study in 2024 using a single postharvest treatment, ‘Dongzao’ fruit began developing mold at the stem-end scars after 5 weeks of cold storage. In 2025, the study was expanded to include multiple postharvest treatments and assessments of physical and biochemical quality attributes. The objectives were to (1) extend the fresh-fruit storage period of ‘Dongzao’ under cold storage and (2) characterize metabolite changes among different metabolite categories to gain insight into the physiological responses of ‘Dongzao’ fruit to prolonged cold storage.

## 2. Results

### 2.1. Fruit Quality and Physiological Parameters

#### 2.1.1. Fruit Vitamin C and Soluble Solids Content Dynamics

**Figure 1:**
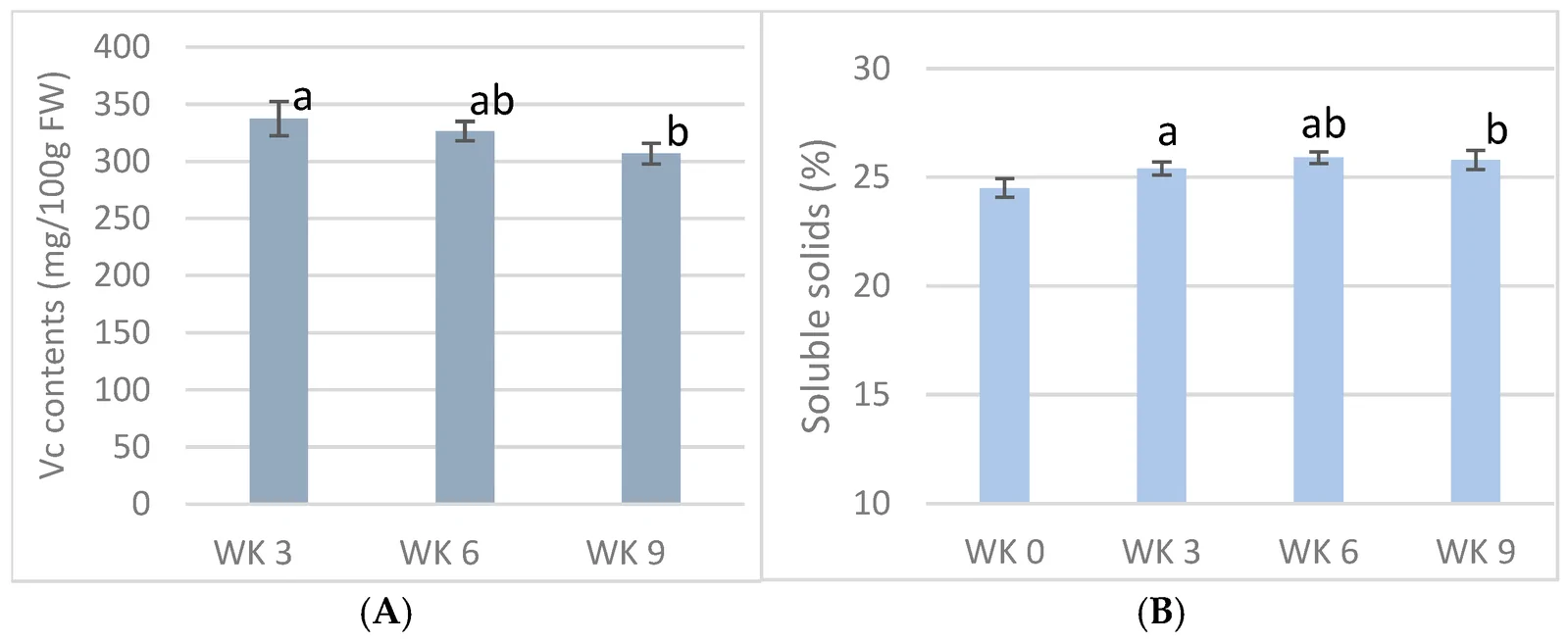
Dongzao fruit vitamin C (**A**) and soluble solids (**B**) content changes during cold storage, WK 0—week 0, WK 3—week 3, WK 6—week 6 and WK 9—week 9. Since there is no significant treatment effect, the number for each sampling date is the average of all treatment samples (mean ± SE). Sampling dates with different letters are significantly different at *p* ≤ 0.05 by Tukey’s honestly significant difference.

Repeated-measures ANOVA showed no significant treatment effect or treatment × sampling week interaction, whereas the sampling week effect was significant. Vitamin C content gradually decreased during cold storage (Figure 1A). At week 3, vitamin C content was significantly higher than at week 9, whereas no significant differences were observed between weeks 3 and 6 or between weeks 6 and 9. On average, vitamin C content decreased from 350 mg/100 g fresh weight (FW) at harvest to 309 mg/100 g FW at week 9, representing an approximately 12% reduction over the 9-week storage period.

At harvest, the soluble solid content (SSC) was 24.5%. No treatment effects on SSC were detected at weeks 3, 6, or 9. However, SSC increased slightly during cold storage, with fruit at week 9 having significantly higher SSC than at week 3 (Figure 1B). No significant differences were observed between weeks 3 and 6 or between weeks 6 and 9 (Figure 1B).

#### 2.1.2. Fruit Rots and Fruit Weight Loss

- Fruit rots

**Figure 2:**
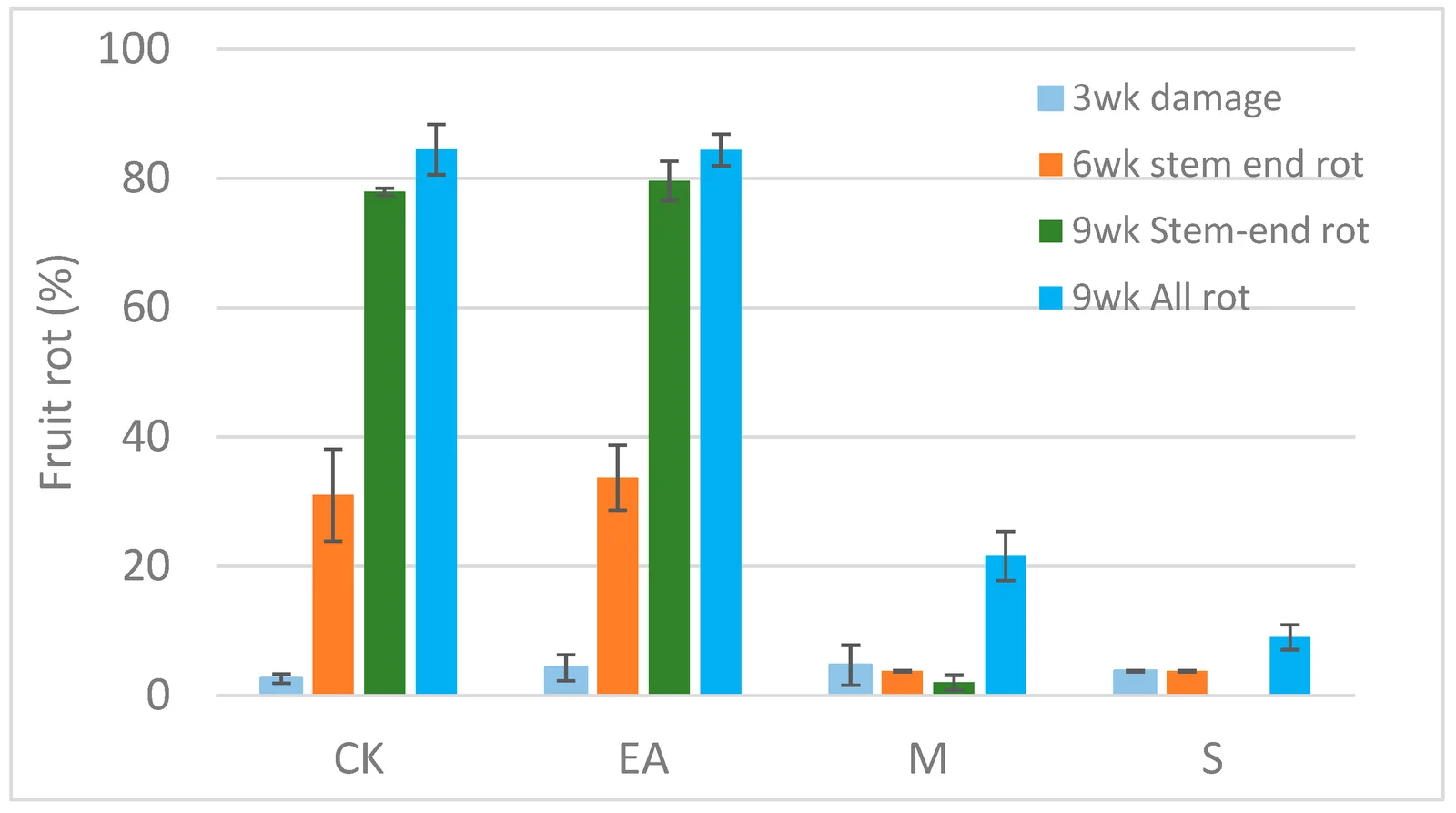
Treatment effects on stem end rot and all rot percentages during Dongzao cold storage. CK—control; EA—ethylene absorbent; M—Mentor fungicide; S—Scholar fungicide. Mean ± SE.

After 6 weeks in cold storage, significant differences in stem-end decay were observed among treatments. Fruit in control and EA treatments exhibited an average of 30.3% and 33.7% stem-end rot/soft decay, respectively. In contrast, fruit treated with either fungicide showed no stem-end rot but had 3.8% decay associated with bruising or shoulder injuries (Figure 2). These injuries were likely attributable to preharvest physical damage, including bruise during picking and a hail event that occurred shortly before harvest.

After 9 weeks in cold storage, ethylene absorbent treatment had stem end decay and total decay incidences comparable to the control with an average decay rate of 84.5% for both CK9 and EA9 (Figure 2). Both fungicide treatments markedly reduced stem end rot and total rot, with Scholar treatment providing better control than Mentor. After 9 weeks of cold storage, Scholar-treated fruit exhibited no stem-end rot with total rot rate of 9.0% (Figure 2).

For the pathogen identification, the pathogen from the fruit rot of Dongzao in cold storage was diagnosed as *Alternaria alternata* by NMSU Plant Diagnostic lab [16].

- Fruit weight losses:

During the 9-week cold storage period, total moisture loss ranged from 1.45% to 2.2% among treatments, with no significant differences observed between treatments.

#### 2.1.3. Fruit Quality Dynamics

**Figure 3:**
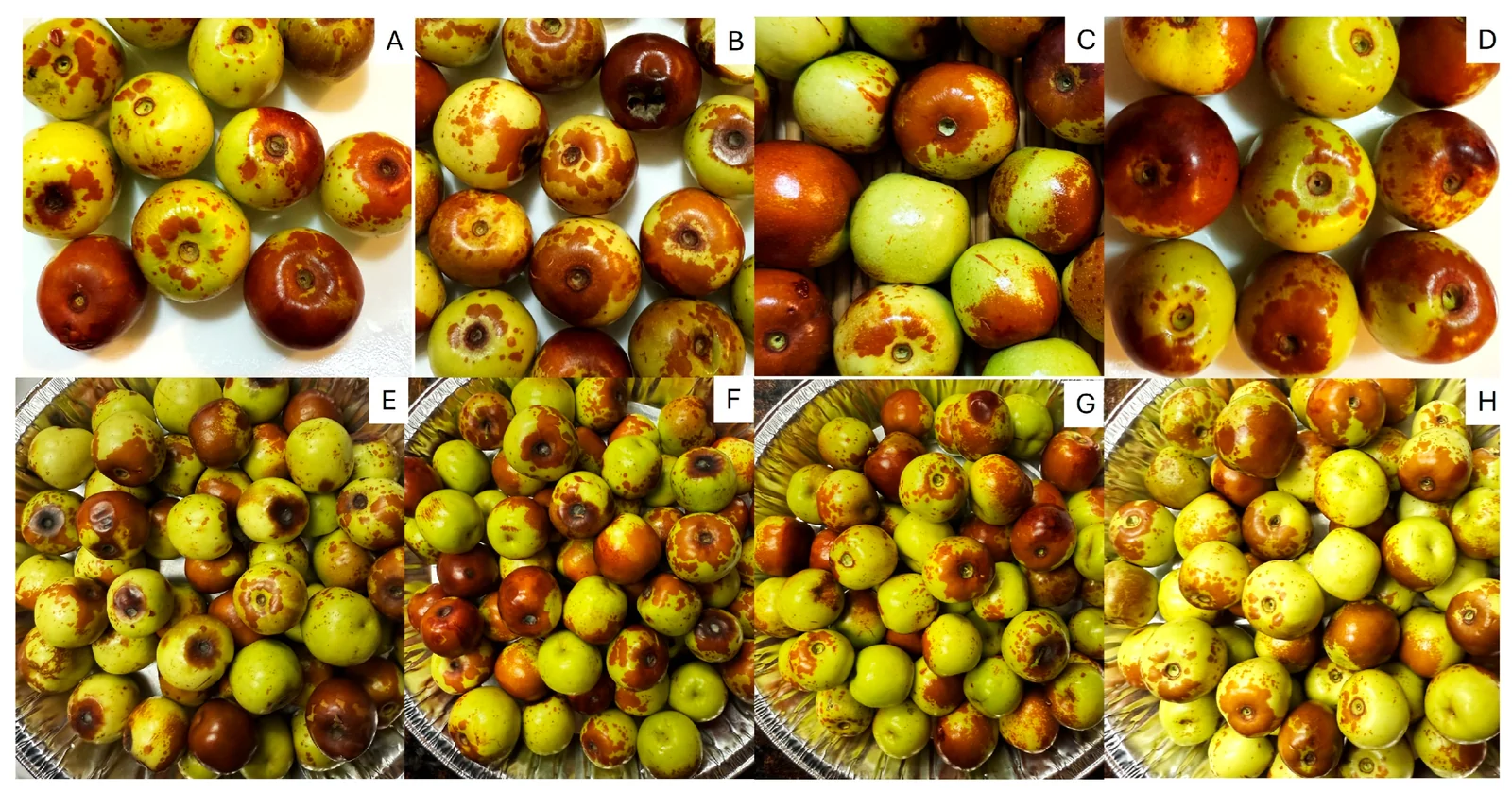
Fruits after 6 or 9 weeks in cold storage of different treatments. (**A**) Control 6 weeks-CK6, (**B**) ethylene absorbent (EA) 6 weeks-EA6, (**C**) Mentor 6 weeks-M6, (**D**) Scholar 6 weeks-S6, (**E**) control 9 weeks-CK9, (**F**) EA 9 weeks-EA9, (**G**) Mentor 9 weeks-M9, (**H**) Scholar 9 weeks-S9.

At week 3, fruit from all treatments had 3–5% damage, primarily associated with bruising and minor preharvest hail injuries. No visible mold was observed at the stem-end scars in any treatment. By week 6, visible mold had developed at the stem ends of fruit in the control and ethylene absorbent (EA) treatments, resulting in decay rates of 31.0% and 33.7%, respectively, primarily stem-end rot (Figure 3A). At this point, fruit in both treatments had lost marketability. In contrast, decay was only 3.8% in both fungicide treatments, with no stem-end rot observed (Figure 3B,C). This decay level was similar to the baseline damage rate observed at week 3 and was primarily associated with preexisting bruising or minor hail injury rather than fungal infection.

After 9 weeks of cold storage, fruit in control and EA treatments had lost marketability, with total decay exceeding 80% (Figure 3D). Fruit treated with Mentor had 2.0% stem-end rot, while the total decay rate reached 21.6%. In contrast, no stem-end rot was observed in the Scholar treatment, and total decay was only 9.0% (Figure 3E). After subtracting the baseline damage rate, the decay rate attributable to storage-related deterioration was approximately 5%, and the fruit remained marketable. These results indicate that Scholar treatment effectively extended the cold-storage life of Dongzao fruit to at least 9 weeks or more than two months.

### 2.2. Metabolomic Analysis

#### 2.2.1. Total Metabolite Detected and Treatment Grouping

**Figure 4:**
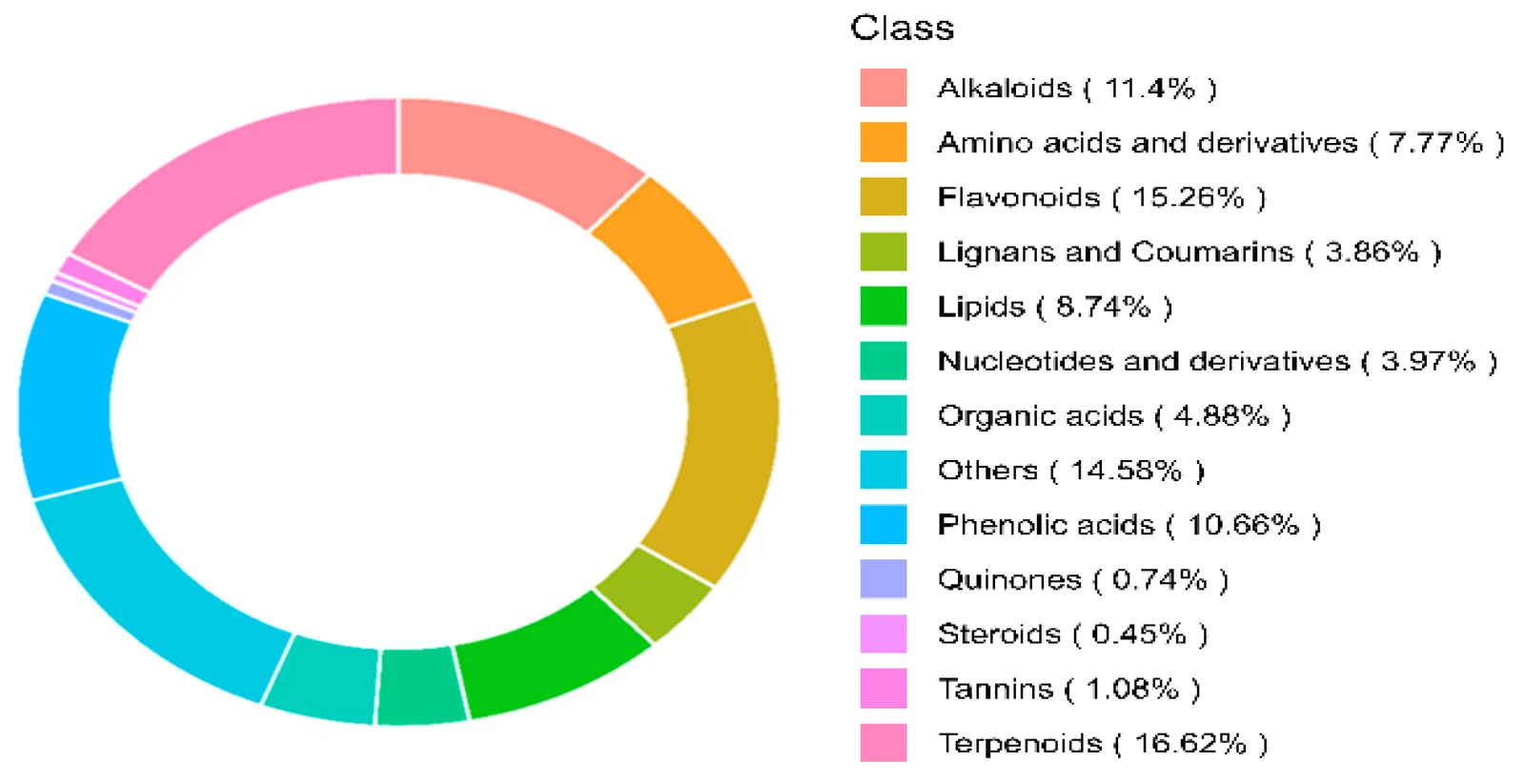
Relative abundance of metabolite categories among 1763 compounds detected in Dongzao cold storage samples in New Mexico, United States in 2025. Categories include Alkaloids, Amino Acids and derivatives, Flavonoids, Ligman and coumarins, Lipids, Nucleotides and derivatives, Organic acids, others, phenolic acids, Quinones, Steroids, Tannins, and Terpenoids.

Based on a commercial database [17], there were 1763 metabolites detected and they were classified into different categories (Figure 4). Among the 1763 metabolites, terpenoids, flavonoids, alkaloids, and phenolic acids accounted for 16.62% (293 metabolites), 15.26% (269 metabolites), 11.4% (201 metabolites) and 10.66% (188 metabolites), respectively, followed by lipids (8.74%, 154 metabolites), amino acids and derivatives (7.77%, 137 metabolites) organic acids (4.88%, 86 metabolites), nucleotides and derivatives (3.97%, 70 metabolites), lignans and coumarins (3.86%, 68 metabolites) and others (14.58%, 257 metabolites) (Figure 4).

**Figure 5:**
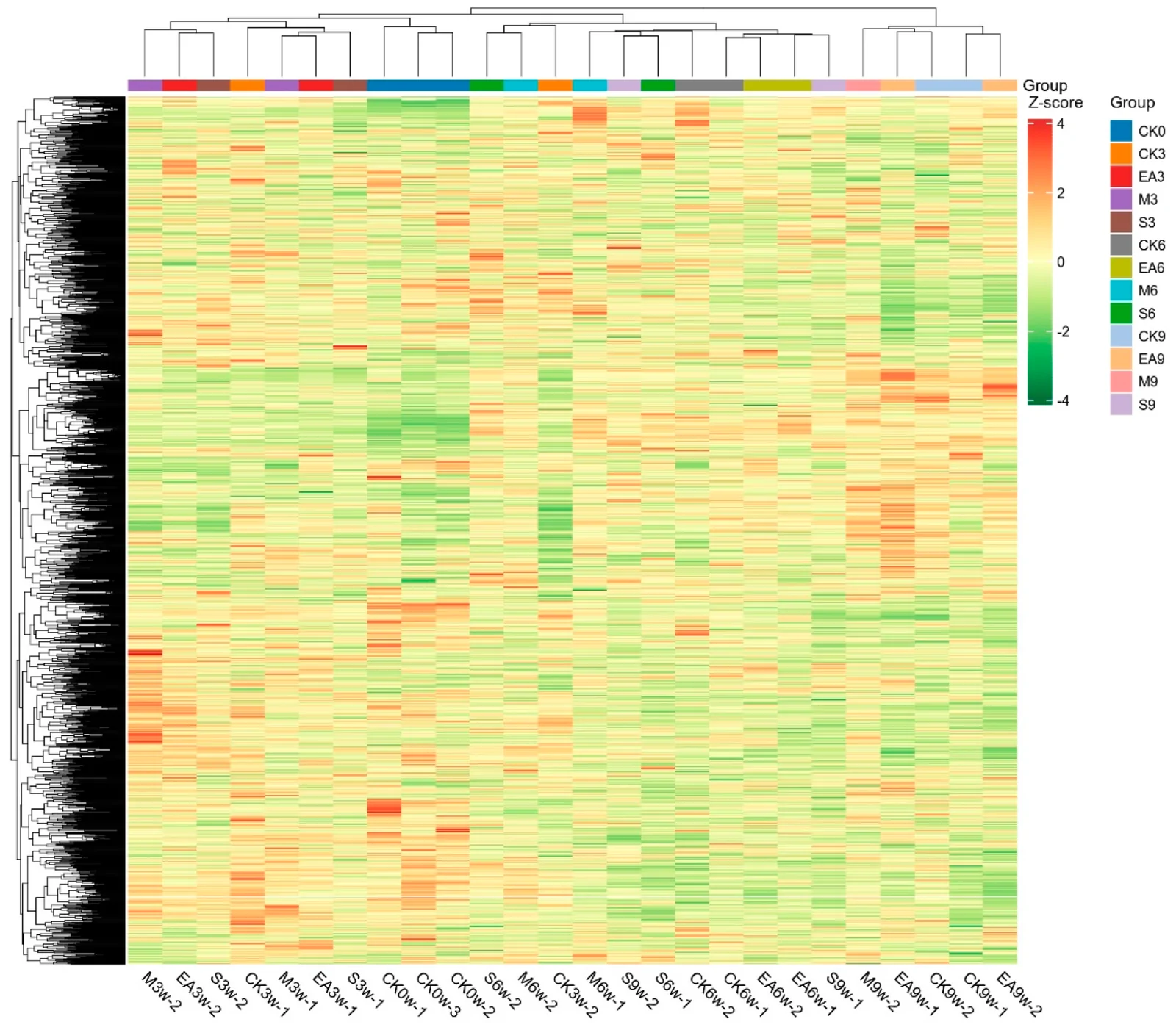
Heatmap of metabolite profiles in ‘Dongzao’ jujube fruit samples subjected to different postharvest treatments and cold storage durations. Hierarchical clustering was performed for both samples (top) and metabolites (left). Colors represent standardized relative metabolite contents, with red indicating higher and green indicating lower relative contents. CK0, CK3, CK6, and CK9 represent control samples at 0, 3, 6, and 9 weeks of cold storage, respectively; EA3, EA6, and EA9 represent ethylene absorbent (EA)−treated samples at 3, 6, and 9 weeks; M3, M6, and M9 represent Mentor−treated samples at 3, 6, and 9 weeks; and S3, S6, and S9 represent Scholar−treated samples at 3, 6, and 9 weeks. Only one sample was available for M9 because the other sample was damaged during shipping.

Sample heatmap grouping results (Figure 5, Figures S1 and S2) indicated that CK0s clustered together, after 3 weeks of cold storage, CK3, EA3, M3 and S3 roughly grouped together except one CK3. CK6, EA6, M6, S6 and S9 grouped together with one CK3. Ck9, EA9 and M9 grouped together further away from other groups. The results corresponded to the fruit decay results. The clustering pattern is consistent with delayed metabolic progression in Scholar-treated fruit. The full metabolite heatmap is in Supplementary Document S1 which will provide a baseline for all metabolites in different treatments and sampling dates and guide future research.

At the same sampling time (weeks 3, 6, or 9), fewer than 100 significantly different metabolites were detected between treatments, except for CK3 vs. CK0 and S9 vs. EA9. Comparisons between week 3 and week 0 identified more differential metabolites than comparisons between weeks 3 and 6 (Table S1). The number of significantly different metabolites increased progressively with storage duration, with more detected in week 6 vs. week 0 than in week 3 vs. week 0, and the greatest number observed in week 9 vs. week 0. In contrast, fewer than 100 differential metabolites were detected between Scholar-treated fruit at week 9 vs. week 6 (S9 vs. S6), suggesting that the Scholar treatment delayed fruit senescence in later part of cold storage, consistent with the visual observations of fruit appearance during cold storage.

#### 2.2.2. 3D Principal Component Analysis

**Figure 6:**
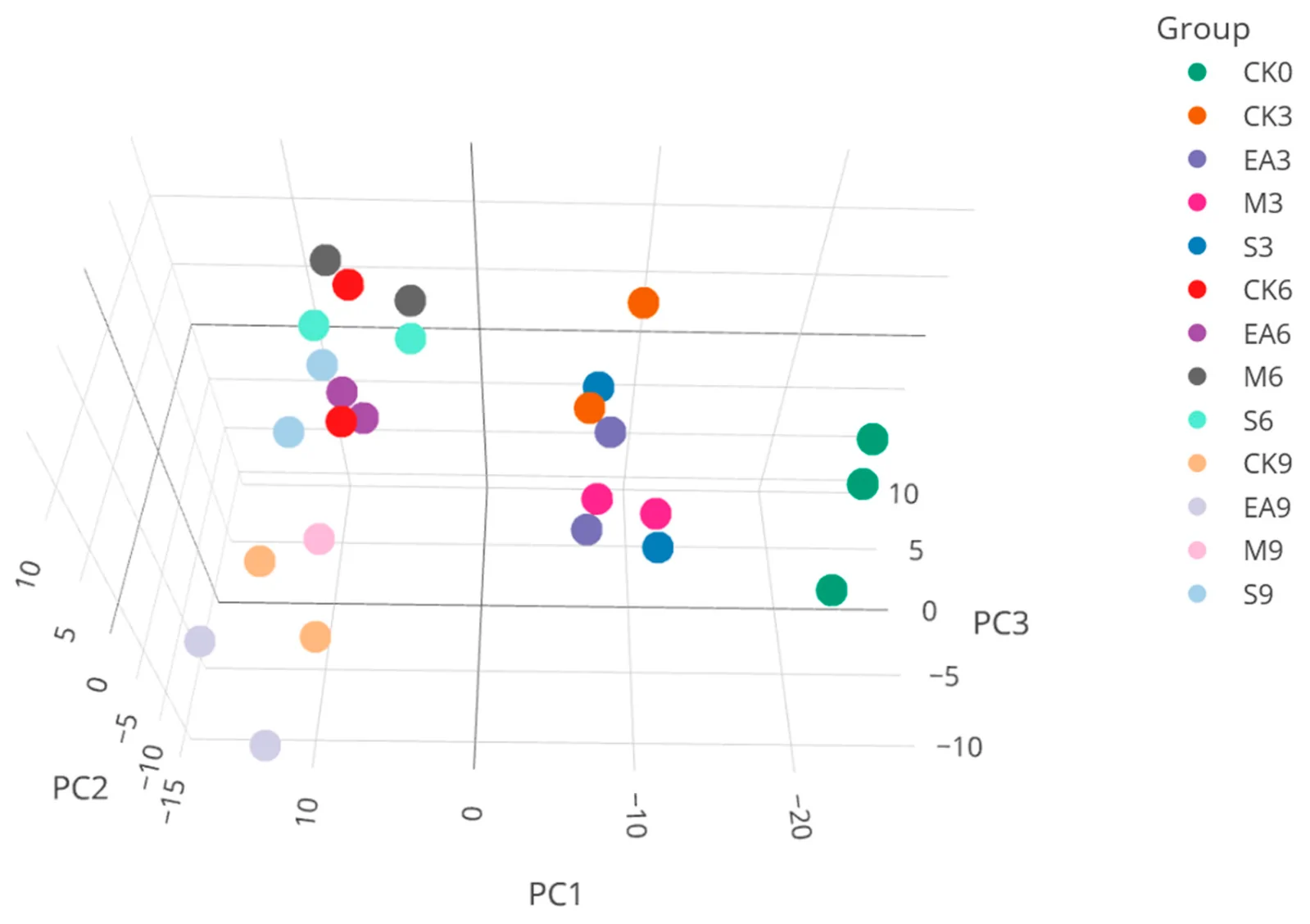
Three-dimension principal component analysis (3D PCA) for all treatments and sampling dates. Each color represents a treatment/sampling date combination, and each dot represents a sample. PC1, PC2, and PC3 denote the first, second, and third principal components and explained 25.03%, 10.54%, and 7.26% of the total variance, respectively, accounting for 42.83% cumulatively. CK0, CK3, CK6, and CK9 represent control samples at 0, 3, 6, and 9 weeks of cold storage, respectively; EA3, EA6, and EA9 represent ethylene absorbent (EA)-treated samples at 3, 6, and 9 weeks; M3, M6, and M9 represent Mentor−treated samples at 3, 6, and 9 weeks; and S3, S6, and S9 represent Scholar−treated samples at 3, 6, and 9 weeks. Only one sample was available for M9 because the other sample was damaged during shipping.

Three-dimensional principal component analysis (3D PCA) showed that the three CK0 samples clustered tightly together. The week 3 samples formed a distinct cluster, followed by the week 6 samples, which clustered with the Scholar week 9 (S9) samples (Figure 6). The remaining week 9 samples (CK9 and EA9) formed a separate cluster. One Mentor week 9 (M9) sample was unavailable, and the remaining M9 sample clustered with the CK9 and EA9 samples but was positioned closer to the week 6 cluster. Those results also indicated that Scholar treatment delayed the fruit senescence process.

#### 2.2.3. Significant Metabolites by Categories

- Lipids changes

The most profound metabolic changes following cold storage were observed in lipids (Table 1). Compared with harvest fruit (CK0), 30, 38, and 42 lipid metabolites were upregulated after 3, 6, and 9 weeks of storage, respectively, with lysophosphatidylcholines (LPCs), lysophosphatidylethanolamines (LPEs), and free fatty acids being the predominant lipid classes (Table 1). In contrast, only 8 and 10 lipid metabolites differed between Scholar-treated and untreated fruit at week 6 (S6 vs. CK6) and week 9 (S9 vs. CK9), respectively. In the S9 vs. CK9 comparison, four fatty acids and one glycerol ester were lower in untreated CK9 fruit, whereas three fatty acids and one glycerol ester were higher in Scholar-treated S9 fruit. These results indicate that low temperature stress in cold storage induced extensive lipid metabolic changes in ‘Dongzao’ fruit beginning as early as week 3 and continuing through week 9. The relatively small number of lipid differences between Scholar-treated and untreated fruit at the same storage duration further suggests that Scholar treatment had a limited effect on the overall lipid turnover/remodeling process.

- Secondary metabolites

Cold storage substantially altered secondary metabolism in ‘Dongzao’ fruit. After 3 weeks of cold storage, the CK3 vs. CK0 comparison showed more upregulated than downregulated alkaloids, flavonoids, phenolic acids, and terpenoids (Table 1). Thereafter, the response shifted, with downregulated secondary metabolites exceeding upregulated metabolites at both 6 and 9 weeks of storage. Flavonoids showed the most pronounced decline, with 36 and 29 flavonoids downregulated at 6 (CK6) and 9 weeks (CK9), respectively, compared with only one and four upregulated compounds. Terpenoids also showed predominantly downward changes, particularly after 9 weeks. Phenolic acids exhibited a more dynamic response, with relatively balanced changes at week 6 but predominantly downregulated metabolites by week 9. In contrast, alkaloids showed a shift toward greater upregulation with prolonged storage. Collectively, these changes indicate substantial remodeling of secondary metabolism during cold storage, with prolonged storage generally associated with a decline in several classes of secondary metabolites, particularly flavonoids.

Scholar treatment produced a different pattern of secondary-metabolite changes during prolonged storage. In the S9 vs. CK0 comparison, eight triterpenes were upregulated and four were downregulated (Table 1). The upregulated triterpenes included uncargenin B, momordicoside aglycone, morolic acid, oleanolic acid, oxyallobetulin, rubiatriol, soyasapogenol E, and unnamed one. A similar pattern was observed at 6 weeks, with nine triterpenes upregulated and two downregulated in S6 vs. CK0. These results suggest that Scholar treatment was associated with the retention or accumulation of several triterpenoid metabolites during cold storage.

Scholar treatment also markedly affected flavonoid metabolism after 9 weeks of cold storage. Ten flavonoids were upregulated in S9 compared with CK9, including phloretin, dihydrokaempferide, dihydrokaempferol-7-O-glucoside, apigenin-8-C-glucoside-7-O-sophoroside, 6-hydroxykaempferol 3-rutinoside, and several glycosylated or malonylated quercetin and isorhamnetin derivatives. The accumulation of these flavonoid metabolites, particularly glycosylated flavonol derivatives, indicates that Scholar treatment modified flavonoid metabolism during prolonged storage.

The detailed up-regulated and down-regulated metabolite heatmaps for CK3 vs. CK0, Ck6 vs. CK0, CK9 vs. CK0, S6 vs. CK0, S9 vs. CK0, S6 vs. CK6, S9 vs. CK9 were in Supplementary Figures S3–S9.

## 3. Discussion

### 3.1. Cold Storage and Fungicide Effects

Based on our 10-year jujube cultivar evaluation trials, ‘Dongzao’ is one of the most promising cultivars for fresh consumption in the United States, and its production acreage is expected to increase as consumer demand develops. However, the delicate nature of ‘Dongzao’ fruit presents a major limitation to its commercialization. The fruit generally maintains acceptable quality for only several days at room temperature and 2–3 weeks under refrigeration. In our preliminary study in 2024, ‘Dongzao’ fruit began developing mold after approximately 5 weeks of cold storage. Previous studies in China have reported storage periods of approximately 2 months, whereas a previous study conducted at the University of California, Davis, in the 1980s just evaluated an unidentified jujube cultivar [11]. Our preliminary observations indicated substantial differences in storability among cultivars; for example, ‘Sherwood’ fruit remained marketable for 8 weeks under 2–4 °C storage conditions [12]. These differences may be related to cultivar-specific fruit characteristics. ‘Dongzao’ has relatively thin skin and a crisp texture, and its pedicels detach readily during normal harvest. The thin skin and exposed stem-end scar may increase susceptibility to bruising during harvest and to stem-end rot during storage, whereas in many other jujube cultivars the pedicel remains attached after harvest.

To our knowledge, this is the first in the United States specifically evaluating the cold storage of ‘Dongzao’ fruit. Our results provide a foundation for further research aimed at extending its postharvest life. The lack of a significant benefit from the ethylene absorbent treatment suggests that ethylene removal alone is not an effective strategy for extending the storage life of ‘Dongzao’. This result is consistent with previous reports characterizing jujube as a nonclimacteric fruit and suggests that factors other than ethylene production or action may play a more important role in postharvest deterioration [18].

An important finding of this study was the potential role of fungicide treatment in extending the cold-storage life of ‘Dongzao’. In previous years, we observed leaf spot disease on jujube plants in New Mexico and identified the causal pathogen as *Alternaria alternata*. At the Los Lunas research plot, leaf spot severity varied by year, apparently depending on precipitation. Although commercial fungicide applications have not been routinely used at this site, the presence of *A. alternata* indicates that this pathogen is established in the production environment. Also at normal picking conditions, Dongzao fruit pedicles detached from the fruit which left an open scar on the stem ends. Fungal infection could be an important factor contributing to postharvest deterioration. In the present study, Scholar treatment substantially reduced fruit decay and extended the marketable storage period of ‘Dongzao’ fruit. Preharvest fungicide applications may therefore be another strategy to reduce pathogen populations before harvest and subsequently lower fruit decay during storage. This approach warrants further investigation under commercial orchard conditions.

Extending the availability of high-quality fresh ‘Dongzao’ fruit by approximately 2 months could have substantial commercial value. A longer storage period would extend the marketing window beyond the short harvest season, reduce the need to market large volumes of fruit immediately after harvest, and potentially allow growers to better target periods of premium market demand. Therefore, improved cold-storage practices, particularly effective management of postharvest fungal decay, could significantly expand and improve the profitability of the U.S. fresh jujube industry.

### 3.2. Metabolite Changes During Cold Storage

Most previous jujube cold storage studies measured selected biochemical compounds (vitamin C, sugars, organic acids, total phenolics, flavonoids, antioxidant activity, and so on), whereas this study shows untargeted metabolite-level changes across lipid and secondary-metabolite classes which provided new insights into metabolic responses and nutrient changes during cold storage and fruit senescence.

### 3.3. Cold Storage Caused Lipid Remodeling

Lipid metabolites showed the most dramatic changes during cold storage, regardless of postharvest treatment. These changes were already evident at the first sampling point (week 3) and continued through week 9, whereas differences among treatments were relatively minor. These results indicate that cold storage induced extensive remodeling of lipid metabolism in ‘Dongzao’ fruit, particularly membrane-associated lipid metabolism, reflecting physiological responses to prolonged low-temperature stress. This finding is consistent with previous studies showing that low-temperature storage induces substantial remodeling of membrane lipids and fatty-acid metabolism in fruit [19,20]. Compared with freshly harvested fruit, 30, 38, and 42 lipid metabolites were upregulated after 3, 6, and 9 weeks of storage, respectively, with lysophosphatidylcholines (LPCs), lysophosphatidylethanolamines (LPEs), and free fatty acids (FFAs) representing the predominant lipid classes. In contrast, only 10 lipid metabolites differed between Scholar-treated and untreated fruit after 9 weeks of storage. The relatively limited differences between S9 and CK9, together with the lower abundance of several fatty acids in S9, suggest that Scholar treatment had a much smaller effect on overall lipid metabolism than prolonged cold storage, but may have modified specific lipid-remodeling processes associated with advanced storage-related senescence.

### 3.4. Cold Storage Caused Broad Changes in Secondary Metabolism

Cold storage substantially altered secondary metabolism in ‘Dongzao’ fruit. At week 3, more alkaloid, flavonoid, phenolic acid, and terpenoid metabolites were upregulated than downregulated relative to freshly harvested fruit. With prolonged storage, flavonoids showed the most pronounced overall decline, followed by terpenoids, whereas phenolic acids exhibited more dynamic patterns of accumulation and depletion (Table 1). Alkaloids, in contrast, showed more upregulated than downregulated metabolites after 9 weeks of storage. These patterns became more evident by week 6 and generally persisted through week 9. Notably, by week 6, untreated fruit had begun to show substantial deterioration, with approximately 30% decay. Previous studies of winter jujube have similarly shown that low-temperature storage alters antioxidant metabolism and the accumulation of phenolic and flavonoid compounds [7,21,22,23,24]. More recently, targeted and spatial metabolomics demonstrated that cold stress in jujube affects polyphenol, proline, glutathione, and lignin-related metabolism, further supporting the view that prolonged cold storage induces broad metabolic reprogramming in jujube fruit [25].

### 3.5. Scholar Treatment and the Secondary-Metabolite Profile

Although cold storage was the primary driver of broad metabolic changes, Scholar treatment was associated with distinct alterations in the secondary-metabolite profile after 9 weeks of storage. Compared with untreated fruit (CK9), Scholar-treated fruit (S9) had 10 upregulated and five downregulated flavonoids, as well as 12 upregulated and five downregulated terpenoids (Table 1). The upregulated flavonoids included several glycosylated and malonylated derivatives of quercetin, kaempferol, isorhamnetin, and apigenin. Notably, many of the upregulated terpenoids were pentacyclic triterpenoids, including ursane- and oleanane-type compounds such as 11-keto-ursolic acid, tomentosolic acid, δ-amyrinone, and several hydroxylated triterpenoid acids. These changes indicate that Scholar treatment was associated with remodeling of specific flavonoid and triterpenoid pathways during prolonged cold storage. Because Scholar-treated fruit also showed substantially less decay and delayed loss of marketability, the altered secondary-metabolite profile may reflect delayed senescence and/or reduced pathogen-related stress. However, metabolomic data alone cannot establish whether these changes resulted directly from fungicide treatment or indirectly from improved physiological condition and reduced microbial deterioration in Scholar-treated fruit.

## 4. Materials and Methods

### 4.1. Plant Material and Preliminary Trial

Dongzao fruits were harvested from the jujube cultivar trial plot at New Mexico State University in Los Lunas. Trees were planted in 2015 and managed under routine cultural practices with flood irrigation [26,27]. In 2024, a preliminary cold storage trial of Dongzao was conducted at 2.2 °C in a walk-in cooler with one treatment. Fruits remained marketable through week 5 but deteriorated afterward, developing severe stem-end rot.

### 4.2. Postharvest Treatment and Experimental Design

In 2025, fruits were harvested on October 2, 2025. Only fruits at the later creamy to early red stage (0–20% red coloration) were used in this study. Fruits (550–580 g/bag) were packed in quart-sized food grade polyethylene zip-lock bags with eight ¼-inch holes per bag. Because of the severe mold issue observed in 2024, two postharvest fungicide treatments, fludioxonil (Scholar SC) and propiconazole (Mentor EC), from Syngenta were included in the study [28,29]. The following four treatments were evaluated: 1. Control (CK), 2. Ethylene absorbent treatment (EA), 3. Fungicide Mentor treatment (M), 4. Fungicide Scholar treatment (S). The storage temperature was 0 ± 0.5 °C in 2025.

Ethylene absorbent packets (WiseFresh 5 g ethylene gas absorber packets) were purchased from Amazon, with one packet placed in each bag. Because packet weights were not identical, the ethylene absorbent packets were removed before sample weighing and then reinserted into the bags after each sampling evaluation.

Scholar and Mentor, both federally registered for postharvest use on jujube in the United States [28,29], were evaluated for their efficacy in extending the cold-storage life of ‘Dongzao’ fruit. Neither product was registered for this use in New Mexico at the time of the study. We obtained both fungicides from Syngenta for research purposes.

For the Mentor and Scholar treatments, fruits were soaked in fungicide solutions at the recommended rates (12.5 mL/L) for 45 and 30 s, respectively. Fruits were then taken out of the solution and air-dried before packaging. Each treatment consisted of 12 bags of fruit (550–580 g). Sampling was conducted at week 0, week 3, week 6, and week 9, with three bags sampled per treatment at each sampling date. At week 0, only three bags were sampled at the start of the study. Samples were then evaluated as follows.

Moisture loss: At each sampling date, each bag was weighed to determine moisture loss.

### 4.3. Fruit Quality Assessment

After each sampling, fruits were visually inspected and manually evaluated for mold development and physical damage.

Soluble solids content: Soluble solids content (SSC, %) was measured using an Atago PAL-1 Digital Pocket Refractometer (Bellevue, WA 98007, USA). A garlic press was used to extract juice from a composite fruit sample from each sample bag.

Vitamin C analysis: Each sample consisted of 6–8 fruits, with two wedges collected from opposite sides of each fruit for a total sample weight of 5 g. Samples were ground with 2% oxalic acid, and the volume was adjusted to 50 mL. After thorough mixing, samples were clarified by centrifugation at 4000 rpm for 10 min at 4 °C using an Eppendorf 5810R (Eppendorf; Hauppauge, NY, USA). The supernatant was titrated with 0.2 mg/mL 2,6-dichlorophenolindophenol [30,31]. Vitamin C content was expressed as mg/100 g fresh weight (FW).

Pathogen identification: During cold storage, once the mold appeared, samples were sent to the NMSU Plant Diagnostic Lab for identification.

### 4.4. Metabolomic Sample Preparation and Analysis

Each treatment included two independent pooled samples (*n* = 2). Although pooling captured contributions from multiple fruits, it prevented assessment of fruit-to-fruit variability. The limited number of independent pools also restricted statistical power and the precision of variance estimates. Therefore, these findings should be considered exploratory. Five fruits were used for each sample, with two wedges collected from opposite sides of each fruit for a total sample weight of 6–7 g. Samples were ground to powder form with liquid nitrogen and stored in a −80 °C freezer. Two samples per treatment at each sampling date were prepared for metabolomic analysis. Samples were sent to a commercial company for metabolomic analysis (Metware Biotechnology LLC, Boston, MA, USA).

#### 4.4.1. Ultra-Performance Liquid Chromatograph-MS Analysis

The samples were submitted to Metware Biotechnology for further preparation, extraction, and metabolomic analysis. The samples were lyophilized using a vacuum (Scientz-100F; Scientz Bio, Ningbo, China). Following this, the samples were ground to powder using a ball-mill grinder (MM400, Retsch; Verder Scientific, Inc., Newtown, PA, USA). For 50 mg of each powdered sample, 1.2 mL of 20 °C precooled 70% methanol was added and then vortexed six times for 30 s every 30 min. Subsequently, the extracts were later centrifuged at 12,000 rpm for 3 min. The supernatant was filtered through a 0.22 µm membrane and stored at 4 °C for further analysis [15].

Samples were analyzed using ultraperformance liquid chromatography (UPLC) (ExionLCTM AD; Sciex, Framingham, MA 01701, USA) and tandem mass spectrometry (MS/MS) (QTRAP 6500; Applied Biosystems, Foster City, CA, USA) under standard operating procedures, as described by Lozada et al. (2023) [32]. UPLC–MS in conjunction with a linear ion trap and a triple quadrupole-linear ion trap mass spectrometer (Q trapVR) (AB6500 Q TRAPVR UPLC/MS/MS system) with an ESI Turbo ion spray interface was used for processing the metabolite extracts. The Analyst 1.6.3 program (Sciex) was used to regulate the cation and anion modes. The operation conditions were as previously stated [33,34].

#### 4.4.2. Qualitative and Quantitative Analysis of Metabolites

Metabolites were identified using an in-house database, the Metware Metabolite Database (Metware Biotechnology Inc., Woburn, MA, USA) [34]. Samples were subjected to semi-quantitative analysis using multiple reaction monitoring (MRM) with a triple quadrupole mass spectrometer. After spectral data acquisition from all samples, peak integration was performed for each detected metabolite. Sample repeatability and analytical stability were evaluated under identical experimental conditions through repeated injections of pooled quality control (QC) samples, which were prepared by combining small aliquots from each sample.

### 4.5. Data Analysis

For the fruit quality related parameters, data were analyzed using ANOVA analysis with Statistix 10 software (Analytical Software, Tallahassee, FL 32312, USA). Means were separated using Tukey’s HSD test at *p* ≤ 0.05.

Commonly used metabolomics data analysis techniques include principal component analysis (PCA), hierarchical clustering analysis (HCA), and orthogonal partial least squares discriminant analysis (OPLS-DA) [15,33,35]. HCA is a multivariate statistical method that groups samples according to similarities in their metabolite profiles, allowing samples with similar characteristics to cluster together.

Metabolite quantification data were normalized using unit variance scaling (UV scaling), and heat maps were generated using the heatmap package in R software 4.1.2 [36]. HCA was subsequently performed to cluster samples based on metabolite composition patterns. PCA was conducted to evaluate differences among treatment groups and to visualize sample variation within and between groups. The prcomp function in R software was used with the parameter scale = TRUE, which applies unit variance scaling for data normalization. Metabolites with log_2_FC (fold change) > 1, VIP > 1, and *p* < 0.05 were set as the threshold of significance.

## 5. Conclusions

Under 0 ± 0.5 °C storage, first-red-stage ‘Dongzao’ fruit remained marketable for approximately 5 weeks without fungicide treatment. Both fungicides reduced decay, with Scholar providing the best protection and extending storage life to 9 weeks without stem-end rot. Cold storage induced substantial lipid remodeling and altered secondary metabolism, with flavonoids showing the greatest decline. Cold storage was the primary driver of metabolomic changes, while Scholar treatment may be associated with changes in flavonoid and triterpenoid profiles after 9 weeks of cold storage, potentially reflecting differences in senescence-related processes. To our knowledge, this is the first study of cold storage and associated metabolomic changes in ‘Dongzao’ fruit in the United States.

## Figures and Tables

**Table 1:** Significant metabolites for selected pairs of treatment and storage time by category. CK0, CK3, CK6, and CK9 represent control week 0, week 3, week 6 and week 9 samples, respectively. S3, S6 and S9 represent Scholar week 3, week 6 and week 9 samples, respectively.

	CK3 vs. CK0		CK6 vs. CK0		S6 vs. CK0		S6 vs. CK6		CK9 vs. CK0		**S9 vs. CK0**		**S9 vs. CK9**	
	Down-Regulated	Up Regulated	Down-Regulated	Up Regulated	Down-Regulated	Up Regulated	Down-Regulated	Up Regulated	Down-Regulated	Up Regulated	Down-Regulated	Up Regulated	Down-Regulated	Up Regulated
Alkaloids	7	9	9	6	8	21	1	7	12	17	10	16	7	1
Amino acids and derivatives	5	3	13	5	6	9		4	5	18	12	7	11	2
Flavonoids	3	8	36	1	24	5	5	11	29	4	26	3	5	10
Lignans and coumarins	2	2	3	5	2	5	1		3	2	10	1		
Lipids	3	30	5	38	9	42	4	4	12	42	9	42	6	4
Free fatty acids	2	14	2	10	5	12	3		7	14	5	12	4	3
Glycerol ester		3	3	3	4	3			5	3	4	2	1	1
LPC		8		15		13	1	1		13		18		
LPE		6		9		13		1		10		9	1	
Sphingolipids	1			1		1		2		2		1		
Nucleotides and derivatives	1	8	5	10	3	10	2	1	2	19	6	14	3	2
Organic acids	4	5	9	4	9	7		1	8	13	10	7	4	
Others	8	15	16	21	20	16	5	7	24	22	35	21	12	6
Phenolic acids	3	11	13	15	16	10	6	3	13	10	25	15	5	2
Quinones	2				1	2				2	2	1		
Tannin			5		1						3		1	
Steroids	1					1				1				
Terpenoids	6	9	21	15	11	13	11	3	30	11	25	15	5	13
Diterpenoids	3	4	3	3	3	3	1		4	4	4	3		
Monoterpenoids		1	2	3	3	2	1		6	1	5	2		1
Sesquiterpenoids	2	1	4	4	2	1	3		6	3	10	1	2	
Terpene								1						1
Triterpene		3	5	5	2	7	6	2	8	3	4	8	2	11
Triterpene saponin	1		3		1		1		6		2	1	1	

## Data Availability

Data will be available upon reasonable request.

## Supplementary Materials

The following supporting information can be downloaded at https://www.mdpi.com/article/10.3390/plants15213370/s1, Figure S1: All treatment and sampling dates of Dongzao cold storage and all metabolites heatmap; Figure S2: Heatmap of all treatments and sampling dates of Dongzao cold storage with clustering for both samples and metabolites; Figure S3: Heatmaps of significant metabolites of Dongzao cold storage for control treatment at week 3 (CK3) vs. control treatment at harvest (CK0); Figure S4: Heatmaps of significant metabolites of Dongzao cold storage for control treatment at week 6 (CK6) vs. control treatment at harvest (CK0); Figure S5: Heatmaps of significant metabolites of Dongzao cold storage for control treatment at week 9 (CK9) vs. control treatment at harvest (CK0); Figure S6: Heatmaps of significant metabolites of Dongzao cold storage for Scholar treatment at week 6 (S6) vs. control treatment at harvest (CK0); Figure S7: Heatmaps of significant metabolites of Dongzao cold storage for Scholar treatment at week 9 (S9) vs. control treatment at harvest (CK0); Figure S8: Heatmaps of significant metabolites of Dongzao cold storage for Scholar treatment at week 6 (S6) vs. control treatment at week 6 (CK6); Figure S9: Heatmaps of significant metabolites of Dongzao cold storage for Scholar treatment at week 9 (S9) vs. control treatment at week 9 (CK9); Table S1: Significant differential metabolites for different pairs of Dongzao cold storage treatments and storage weeks. If A vs. B is down-regulated, B has more metabolite than A; if up-regulated, A has more metabolite than B.

## References

[1] Guo Y., Shan G. (2010). The Chinese Jujube.

[2] Yao S. (2023). Jujube Cultivars in New Mexico. New Mexico Cooperative Extension Service Publication H340. https://pubs.nmsu.edu/_h/H340.pdf.

[3] Liu M., Wang M. (2019). Germplasm Resources of Chinese Jujube.

[4] Deng J., Han C., Wang W., Cao J., Zhou Z., Zhao G., Huang X. (2026). Effects of different storage temperatures on the shelf life and quality characteristics of winter jujubes. Sci. Hortic..

[5] Jin T., Dai C., Xu Y., Chen Y., Xu Q., Wu Z. (2022). Applying Cold Atmospheric Plasma to Preserve the Postharvest Qualities of Winter Jujube (*Ziziphus jujuba* Mill. cv. Dongzao) During Cold Storage. Front. Nutr..

[6] Ozturk B., Aglar E., Gun S., Karakaya O. (2020). Change of Fruit Quality Properties of Jujube Fruit (*Ziziphus jujuba*) without Stalk and with Stalk during Cold Storage. Int. J. Fruit. Sci..

[7] Sang Y., Yang W., Liu Y., Zhang W., Guo T., Shen P., Tang Y., Guo M., Chen G. (2022). Influences of low temperature on the postharvest quality and antioxidant capacity of winter jujube (*Zizyphus jujuba* Mill. cv. Dongzao). LWT-Food Sci. Technol..

[8] Tang W., Xiang B., Cao Y., Wu C., Zhang C., Lin M., Du P. (2025). 1-MCP maintains postharvest quality in winter jujube during low-temperature storage by regulating energy and sugar metabolism and enhancing antioxidant capacity. Front. Plant Sci..

[9] Yang L., Liu J., Wang X., Wang R., Ren F., Zhang Q., Shan Y., Ding S. (2019). Characterization of Volatile Component Changes in Jujube Fruits during Cold Storage by Using Headspace-Gas Chromatography-Ion Mobility Spectrometry. Molecules.

[10] Zhao Y., Zhu X., Hou Y., Pan Y., Xin L., Li Z. (2021). Effects of harvest maturity stage on postharvest quality of winter jujube (*Zizyphus jujuba* Mill. cv. Dongzao) fruit during cold storage. Sci. Hortic..

[11] Kader A.A., Li Y., Chordas A. (1982). Postharvest respiration, ethylene production, and compositional changes of Chinese jujube fruits. HortScience.

[12] Yao S., Heyduck R., Huang J. (2015). Preliminary report on jujube cold storage and nutrient changes. HortScience.

[13] Alseekh S., Aharoni A., Brotman Y., Contrepois K., D’Auria J., Ewald J., Ewald J.C., Fraser P.D., Giavalisco P., Hall R.D. (2021). Mass spectrometry-based metabolomics: A guide for annotation, quantification and best reporting practices. Nat. Methods.

[14] Sapkota D., Holguin F.O., Salmasi S.Z., Yao S. (2025). Fruit and seed metabolomic profiles provide insights into the utilization of sour jujube germplasm. J. Am. Soc. Hortic. Sci..

[15] Yao S., Sapkota D., Hungerford J.A., Kersten R.K. (2023). Jujube Fruit Metabolomic Profiles Reveal Cultivar Differences and Function as Cultivar Fingerprints. Plants.

[16] Lujan P., Dura S., Yao S., Barraza J. First Report of jujube leaf spot and postharvest fruit rot caused by *Alternaria alternata* in New Mexico (Abstract). Proceedings of the International Horticultural Congress 2026.

[17] Wishart D.S., Knox C., Guo A.C., Eisner R., Young N., Gautam B., Hau D.D., Psychogios N., Dong E., Bouatra S. (2009). HMDB: A knowledgebase for the human metabolome. Nucleic Acids Res..

[18] Liu M., Wang J., Wang L., Liu P., Zhao J., Zhao Z., Yao S., Stanica F., Liu Z., Wang L. (2020). The historical and current research progresses of jujube- a super fruit for the future. Hortic. Res..

[19] Li Q., Lin H., Lin H., Lin M., Wang H., Wei W., Chen J., Li W., Shao X., Fan Z. (2023). The metabolism of membrane lipid participates in the occurrence of chilling injury in cold-stored banana fruit. Food Res. Int..

[20] Song C., Wang K., Xiao X., Liu Q., Yang M., Li X., Feng Y., Li S., Shi L., Chen W. (2022). Membrane lipid metabolism influences chilling injury during cold storage of peach fruit. Food Res. Int..

[21] Zhao Y., Zhu X., Hou Y., Wang X., Li X. (2019). Effects of nitric oxide fumigation treatment on retarding cell wall degradation and delaying softening of winter jujube (*Ziziphus jujuba* Mill. Cv. Dongzao) fruit during cold storage. Postharvest Biol. Technol..

[22] Guo X., Liu Q., Du J., Guo Y., Hu X., Yu J., Bai J., Li X., Kou L. (2022). X-rays irradiation affects flavonoid synthesis and delays reddening of winter jujube (*Zizyphus jujuba* Mill. cv. Dalidongzao) during cold storage. Postharvest Biol. Technol..

[23] Islam A., Acıkalın R., Ozturk B., Aglar E., Kaiser C. (2022). Combined effects of Aloe vera gel and modified atmosphere packaging treatments on fruit quality traits and bioactive compounds of jujube (*Ziziphus jujuba* Mill.) fruit during cold storage and shelf life. Postharvest Biol. Technol..

[24] Ozturk B., Yildiz M., Yildiz K., Gun S. (2020). Maintaining the postharvest quality and bioactive compounds of jujube (*Ziziphus jujuba* Mill. cv. ‘Li’) fruit by applying 1-methylcyclopropene. Sci. Hortic..

[25] Niu C., Guo T., Xu W., Gao Y., Liu L., Liu J., Ban Z., Wu Z., Jiang Y. (2025). Targeted and spatial metabolomics unveil how brassinolide enhances polyphenol and proline metabolism in cold-stressed jujube fruit. Postharvest Biol. Technol..

[26] Yao S., Heyduck R., Guldan S. (2019). Early performance of jujube fresh eating cultivars in the southwestern United States. HortScience.

[27] Yao S., Heyduck R., Guldan S.J., Sapkota G. (2020). Early performance of jujube drying and multipurpose cultivars in the southwestern United States. HortScience.

[28] Syngenta USA (2020). Mentor EC Label. https://www.syngenta-us.com/current-label/mentor_ec.

[29] Syngenta USA (2022). Scholar SC Label. https://www.syngenta-us.com/current-label/scholar_sc.

[30] Bessey O. (1933). The distribution of vitamin C in plant and animal tissues, and its determination. J. Biol. Chem..

[31] Huang J., Heyduck R., Richins R., VanLeeuwen D., O’Connell M.A., Yao S. (2017). Jujube Cultivar Vitamin C Profile and Nutrient Dynamics during Maturation. HortScience.

[32] Lozada D.N., Pulicherla S.R., Holguin F.O. (2023). Widely Targeted Metabolomics Reveals Metabolite Diversity in Jalapeño and Serrano Chile Peppers (*Capsicum annuum* L.). Metabolites.

[33] Cao H., Ji Y., Li S., Lu L., Tian M., Yang W., Li H. (2019). Extensive metabolic profiles of leaves and stems from the medicinal plant Dendrobium officinale Kimura et Migo. Metabolites.

[34] Chen W., Gong L., Guo Z., Wang W., Zhang H., Liu X., Yu S., Xiong L., Luo J. (2013). A novel integrated method for large-scale detection, identification, and quantification of widely targeted metabolites: Application in the study of rice metabolomics. Mol. Plant.

[35] Wang S., Tu H., Wan J., Chen W., Liu X., Luo J., Xu J., Zhang H. (2016). Spatio-temporal distribution and natural variation of metabolites in citrus fruits. Food Chem..

[36] Chong J., Xia J. (2018). MetaboAnalystR: An R package for flexible and reproducible analysis of metabolomics data. Bioinformatics.

