# Supplementary material for: Lipid and Secondary Metabolite Profile Changes During Cold Storage of Dongzao (Ziziphus jujuba Mill. cv. Dongzao): Responses to Postharvest Treatments and Implications for Marketability
Source: Plants (Basel). 2026 Nov 1;15(21):3370. doi: 10.3390/plants15213370 (PMC13766002; doi:10.3390/plants15213370)
Supplement: Supplementary file 1 [file plants-15-03370-s001.zip › plants-4600903-supplementary.pdf]

"Jujube Dongzao cold storage and metabolomic changes" supplementary documents:

Shengrui Yao[1,2] and F. Omar Holguin[1]

1.Department of Plant and Environmental Sciences, New Mexico State University, Las Cruces, NM 88003, USA

2.Sustainable Agriculture Science Center at Alcalde, New Mexico State University, Alcalde, NM 87511, USA

Figure	Description	Page
Figure S1	All treatment and sampling dates of Dongzao cold storage and all metabolites heatmap	2
Figure S2	Heatmap of all treatments and sampling dates of Dongzao cold storage with clustering for both samples and metabolites	3
Figure S3	Heatmaps of significant metabolites of Dongzao cold storage for control treatment at week 3 (CK3) vs. control treatment at harvest (CK0).	4
Figure S4	Heatmaps of significant metabolites of Dongzao cold storage for control treatment at week 6 (CK6) vs. control treatment at harvest (CK0).	5
Figure S5	Heatmaps of significant metabolites of Dongzao cold storage for control treatment at week 9 (CK9) vs. control treatment at harvest (CK0).	6
Figure S6	Heatmaps of significant metabolites of Dongzao cold storage for Scholar treatment at week 6 (S6) vs. control treatment at harvest (CK0).	7
Figure S7	Heatmaps of significant metabolites of Dongzao cold storage for Scholar treatment at week 9 (S9) vs. control treatment at harvest (CK0).	8
Figure S8	Heatmaps of significant metabolites of Dongzao cold storage for Scholar treatment at week 6 (S6) vs. control treatment at week 6 (CK6).	9
Figure S9	Heatmaps of significant metabolites of Dongzao cold storage for Scholar treatment at week 9 (S9) vs. control treatment at week 9 (CK9).	10
Table S1	Significant differential metabolites for different pairs of Dongzao cold storage treatments and storage weeks. If A vs. B is down-regulated, B has more metabolite than A; if up-regulated, A has more metabolite than B.	11

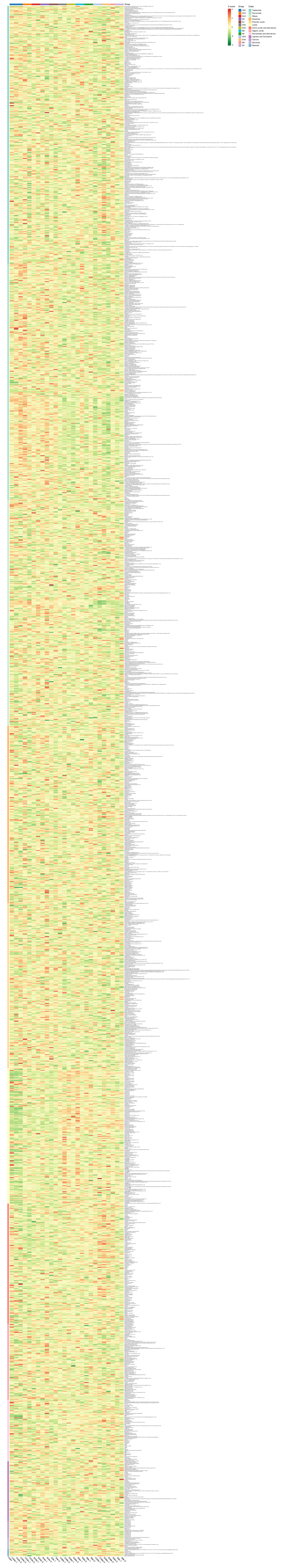

Group
Z-score
4
2
0
-2
-4
Group
CK0
CK3
EA3
M3
S3
CK6
EA6
M6
S6
CK9
EA9
M9
S9
Class
Terpenoids
Flavonoids
Others
Alkaloids
Phenolic acids
Lipids
Amino acids and derivatives
Organic acids
Nucleotides and derivatives
Lignans and Coumarins
Tannins
Quinones
Steroids

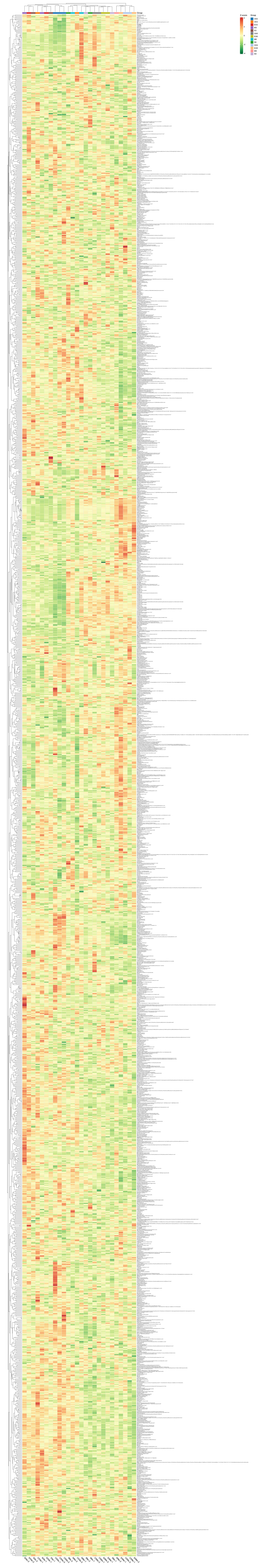
Group
Z-score
4
2
0
-2
-4
Group
CK0
CK3
EA3
M3
S3
CK6
EA6
M6
S6
CK9
EA9
M9
S9

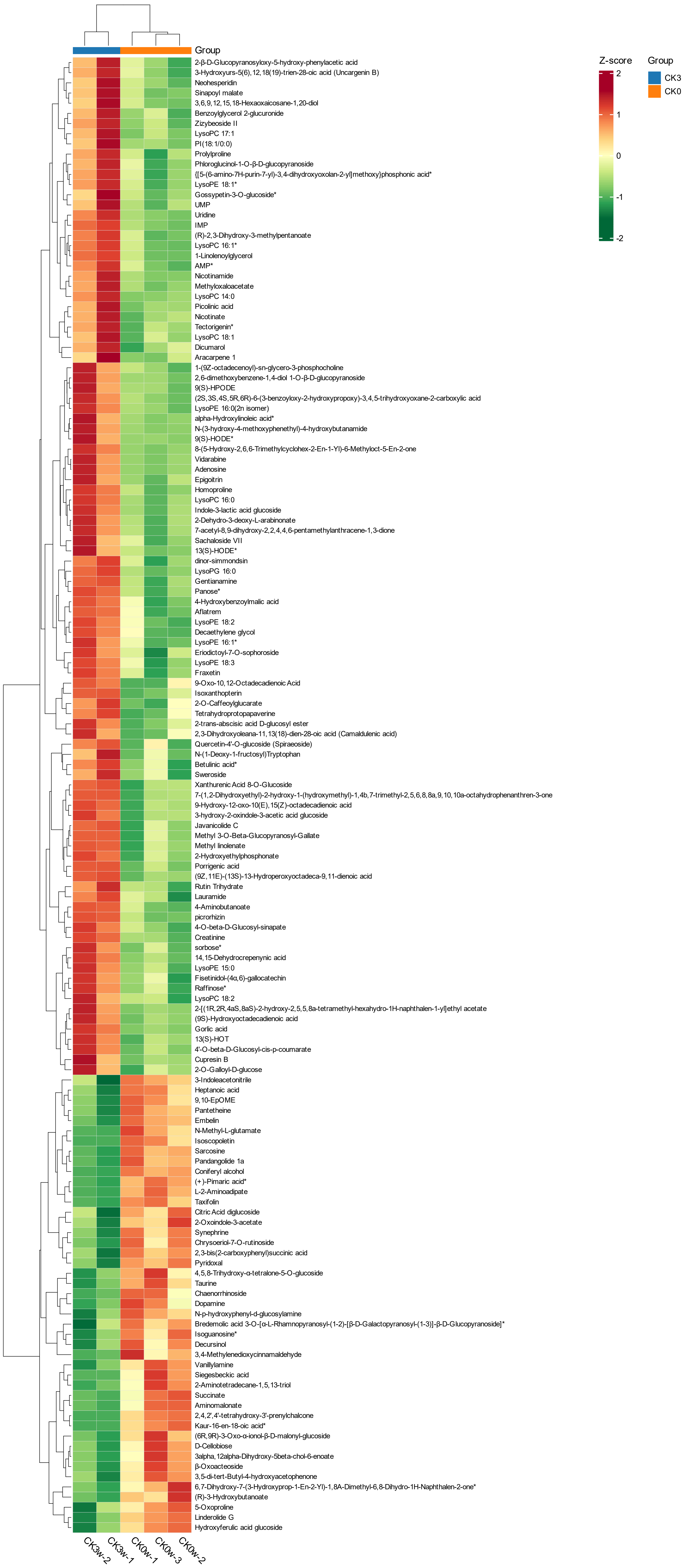

Group
Z-score
2
1
0
-1
-2
Group
CK3
CK0
2-β-D-Glucopyranosyloxy-5-hydroxy-phenylacetic acid
3-Hydroxyurs-5(6),12,18(19)-trien-28-oic acid (Uncargenin B)
Neohesperidin
Sinapoyl malate
3,6,9,12,15,18-Hexaoxaicosane-1,20-diol
Benzoylglycerol 2-glucuronide
Zizybeoside II
LysoPC 17:1
PI(18:1/0:0)
Prolylproline
Phloroglucinol-1-O-β-D-glucopyranoside
{[5-(6-amino-7H-purin-7-yl)-3,4-dihydroxyoxolan-2-yl]methoxy}phosphonic acid*
LysoPE 18:1*
Gossypetin-3-O-glucoside*
UMP
Uridine
IMP
(R)-2,3-Dihydroxy-3-methylpentanoate
LysoPC 16:1*
1-Linolenoylglycerol
AMP*
Nicotinamide
Methyloxaloacetate
LysoPC 14:0
Picolinic acid
Nicotinate
Tectorigenin*
LysoPC 18:1
Dicumarol
Aracarpene 1
1-(9Z-octadecenoyl)-sn-glycero-3-phosphocholine
2,6-dimethoxybenzene-1,4-diol 1-O-β-D-glucopyranoside
9(S)-HPODE
(2S,3S,4S,5R,6R)-6-(3-benzoyloxy-2-hydroxypropoxy)-3,4,5-trihydroxyoxane-2-carboxylic acid
LysoPE 16:0(2n isomer)
alpha-Hydroxylinoleic acid*
N-(3-hydroxy-4-methoxyphenethyl)-4-hydroxybutanamide
9(S)-HODE*
8-(5-Hydroxy-2,6,6-Trimethylcyclohex-2-En-1-Yl)-6-Methyloct-5-En-2-one
Vidarabine
Adenosine
Epigoitrin
Homoproline
LysoPC 16:0
Indole-3-lactic acid glucoside
2-Dehydro-3-deoxy-L-arabinonate
7-acetyl-8,9-dihydroxy-2,2,4,4,6-pentamethylanthracene-1,3-dione
Sachaloside VII
13(S)-HODE*
dinor-simmondsin
LysoPG 16:0
Gentianamine
Panose*
4-Hydroxybenzoylmalic acid
Aflatrem
LysoPE 18:2
Decaethylene glycol
LysoPE 16:1*
Eriodictyol-7-O-sophoroside
LysoPE 18:3
Fraxetin
9-Oxo-10,12-Octadecadienoic Acid
Isoxanthopterin
2-O-Caffeoylglucarate
Tetrahydroprotopapaverine
2-trans-abscisic acid D-glucosyl ester
2,3-Dihydroxyoleana-11,13(18)-dien-28-oic acid (Camaldulenic acid)
Quercetin-4'-O-glucoside (Spiraeoside)
N-(1-Deoxy-1-fructosyl)Tryptophan
Betulinic acid*
Sweroside
Xanthurenic Acid 8-O-Glucoside
7-(1,2-Dihydroxyethyl)-2-hydroxy-1-(hydroxymethyl)-1,4b,7-trimethyl-2,5,6,8,8a,9,10,10a-octahydrophenanthren-3-one
9-Hydroxy-12-oxo-10(E),15(Z)-octadecadienoic acid
3-hydroxy-2-oxindole-3-acetic acid glucoside
Javanicolide C
Methyl 3-O-Beta-Glucopyranosyl-Gallate
Methyl linolenate
2-Hydroxyethylphosphonate
Porrigenic acid
(9Z,11E)-(13S)-13-Hydroperoxyoctadeca-9,11-dienoic acid
Rutin Trihydrate
Lauramide
4-Aminobutanoate
picrorhizin
4-O-beta-D-Glucosyl-sinapate
Creatinine
sorbose*
14,15-Dehydrocrepenynic acid
LysoPE 15:0
Fisetinidol-(4α,6)-gallocatechin
Raffinose*
LysoPC 18:2
2-[(1R,2R,4aS,8aS)-2-hydroxy-2,5,5,8a-tetramethyl-hexahydro-1H-naphthalen-1-yl]ethyl acetate
(9S)-Hydroxyoctadecadienoic acid
Gorlic acid
13(S)-HOT
4'-O-beta-D-Glucosyl-cis-p-coumarate
Cupresin B
2-O-Galloyl-D-glucose
3-Indoleacetonitrile
Heptanoic acid
9,10-EpOME
Pantetheine
Embelin
N-Methyl-L-glutamate
Isoscopoletin
Sarcosine
Pandangolide 1a
Coniferyl alcohol
(+)-Pimaric acid*
L-2-Aminoadipate
Taxifolin
Citric Acid diglucoside
2-Oxoindole-3-acetate
Synephrine
Chrysoeriol-7-O-rutinoside
2,3-bis(2-carboxyphenyl)succinic acid
Pyridoxal
4,5,8-Trihydroxy-α-tetralone-5-O-glucoside
Taurine
Chaenorrhinoside
Dopamine
N-p-hydroxyphenyl-d-glucosylamine
Bredemolic acid 3-O-[α-L-Rhamnopyranosyl-(1-2)-[β-D-Galactopyranosyl-(1-3)]-β-D-Glucopyranoside]*
Isoguanosine*
Decursinol
3,4-Methylenedioxycinnamaldehyde
Vanillylamine
Siegesbeckic acid
2-Aminotetradecane-1,5,13-triol
Succinate
Aminomalonate
2,4,2',4'-tetrahydroxy-3'-prenylchalcone
Kaur-16-en-18-oic acid*
(6R,9R)-3-Oxo-α-ionol-β-D-malonyl-glucoside
D-Cellobiose
3alpha,12alpha-Dihydroxy-5beta-chol-6-enoate
β-Oxoacteoside
3,5-di-tert-Butyl-4-hydroxyacetophenone
6,7-Dihydroxy-7-(3-Hydroxyprop-1-En-2-Yl)-1,8A-Dimethyl-6,8-Dihydro-1H-Naphthalen-2-one*
(R)-3-Hydroxybutanoate
5-Oxoproline
Linderolide G
Hydroxyferulic acid glucoside
CK3w-2
CK3w-1
CK0w-1
CK0w-3
CK0w-2

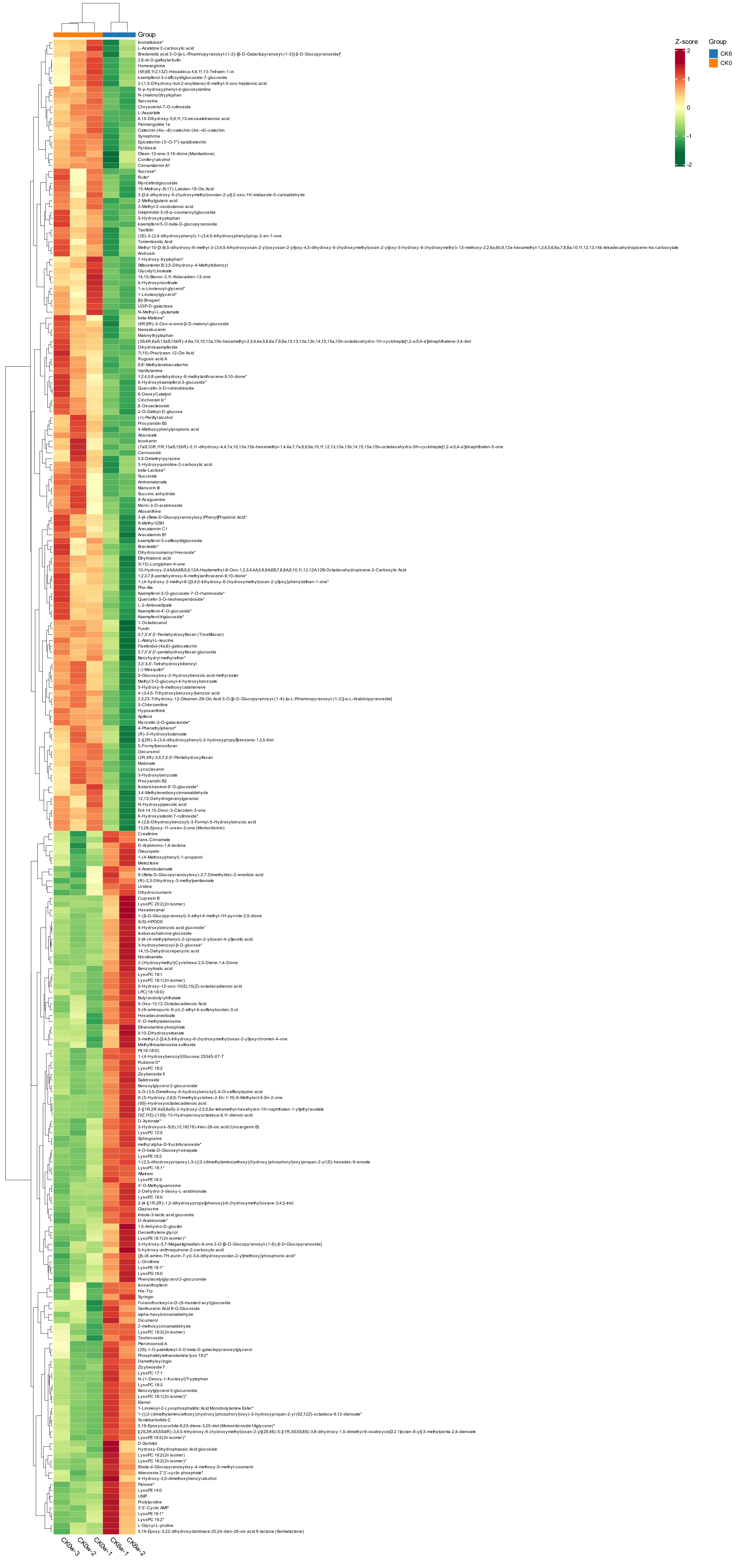

Group
Z-score
2
1
0
-1
-2
Group
CK6
CK0
Isomaltulose*
L-Azetidine 2-carboxylic acid
Bredemolic acid 3-O-[α-L-Rhamnopyranosyl-(1-2)-[β-D-Galactopyranosyl-(1-3)]-β-D-Glucopyranoside]*
2,6-di-O-galloylarbutin
Homoarginine
(4E,6E,11Z,13Z)-Hexadeca-4,6,11,13-Tetraen-1-ol
kaempferol-3-caffeoyldiglucoside-7-glucoside
2-(1,3-Dihydroxy-but-2-enylidene)-6-methyl-3-oxo-heptanoic acid
N-p-hydroxyphenyl-d-glucosylamine
N-(malonyl)tryptophan
Sarcosine
Chrysoeriol-7-O-rutinoside
L-Aspartate
8,15-Dihydroxy-5,9,11,13-eicosatetraenoic acid
Pandangolide 1a
Catechin-(4α→8)-catechin-(4α→6)-catechin
Synephrine
Epicatechin-(3'-O-7'')-epiafzelechin
Pyridoxal
Olean-12-ene-3,16-dione (Maniladione)
Coniferyl alcohol
Cinnamtannin A1
Sucrose*
Rutin*
Myricetindiglucoside
15-Methoxy-8(17)-Labden-19-Oic Acid
3-[3,4-dihydroxy-5-(hydroxymethyl)oxolan-2-yl]-2-oxo-1H-imidazole-5-carbaldehyde
2-Methylglutaric acid
3-Methyl-2-oxobutanoic acid
Delphinidin 3-(6-p-coumaroyl)glucoside
5-Hydroxytryptophan
kaempferol 5-O-beta-D-glucopyranoside
Taxifolin
(2E)-3-(2,4-dihydroxyphenyl)-1-(3,4,5-trihydroxyphenyl)prop-2-en-1-one
Tomentosolic Acid
Methyl 10-[3-[4,5-dihydroxy-6-methyl-3-(3,4,5-trihydroxyoxan-2-yl)oxyoxan-2-yl]oxy-4,5-dihydroxy-6-(hydroxymethyl)oxan-2-yl]oxy-5-hydroxy-9-(hydroxymethyl)-13-methoxy-2,2,6a,6b,9,12a-hexamethyl-1,3,4,5,6,6a,7,8,8a,10,11,12,13,14b-tetradecahydropicene-4a-carboxylate
Androsin
7-Hydroxy-tryptophan*
Stilbostemin B;3,5-Dihydroxy-4-Methylbibenzyl
Glycidyl Linoleate
14,15-Bisnor-3,11-Kolavadien-13-one
6-Hydroxynicotinate
1-α-Linolenoyl-glycerol*
1-Linoleoylglycerol*
[6]-Shogaol
UDP-D-galactose
N-Methyl-L-glutamate
beta-Maltose*
(6R,9R)-3-Oxo-α-ionol-β-D-malonyl-glucoside
Neosakuranin
Malonyltryptophan
(3S,4R,6aS,13aS,15bR)-4,6a,10,10,13a,15b-hexamethyl-2,3,4,4a,5,6,6a,7,9,9a,10,13,13a,13b,14,15,15a,15b-octadecahydro-1H-cyclohepta[1,2-a:5,4-a']dinaphthalene-3,4-diol
Dihydrokaempferide
7(15)-Prezizaen-12-Oic Acid
Rugosic acid A
8,8'-Methylenebiscatechin
Vanillylamine
1,2,4,5,8-pentahydroxy-6-methylanthracene-9,10-dione*
6-Hydroxykaempferol 3-glucoside*
Quercetin-3-O-robinobioside
6-DeoxyCatalpol
Cinchonain Ic*
β-Oxoacteoside
2-O-Galloyl-D-glucose
(+)-Perillyl alcohol
Procyanidin B3
4-Methoxyphenylpropionic acid
Abscisate
Isookanin
(7aS,10R,11R,13aS,15bR)-3,11-dihydroxy-4,4,7a,10,13a,15b-hexamethyl-1,4,4a,7,7a,8,9,9a,10,11,12,13,13a,13b,14,15,15a,15b-octadecahydro-5H-cyclohepta[1,2-a:5,4-a']dinaphthalen-5-one
Cernuoside
2,5-Dimethylpyrazine
3-Hydroxyquinoline-2-carboxylic acid
beta-Lactose*
Succinate
Aminomalonate
Mansorin III
Succinic anhydride
8-Azaguanine
Morin-3-O-arabinoside
Alloxanthine
3-[4-(Beta-D-Glucopyranosyloxy)Phenyl]Propionic Acid*
S-Methyl GSH
Arecatannin C1
Arecatannin B1
kaempferol-3-caffeoyldiglucoside
Bracteatin*
Dihydrocoumaroyl Hexoside*
Ethylmalonic acid
3(15)-Longipinen-4-one
10-Hydroxy-2,4A,6A,6B,9,9,12A-Heptamethyl-8-Oxo-1,2,3,4,4A,5,6,6A,6B,7,8,8A,9,10,11,12,12A,12B-Octadecahydropicene-2-Carboxylic Acid
1,2,3,7,8-pentahydroxy-6-methylanthracene-9,10-dione*
1-(4-hydroxy-2-methyl-6-[3,4,5-trihydroxy-6-(hydroxymethyl)oxan-2-yl]oxy]phenyl)ethan-1-one*
Phe-Ala
Kaempferol-3-O-glucoside-7-O-rhamnoside*
Quercetin-3-O-neohesperidoside*
L-2-Aminoadipate
Kaempferol-4'-O-glucoside*
Kaempferol triglucoside*
1-Octadecanol
Fustin
5,7,3',4',5'-Pentahydroxyflavan (Tricetiflavan)
L-Alanyl-L-leucine
Fisetinidol-(4α,6)-gallocatechin
5,7,3',4',5'-pentahydroxyflavan glucoside
Benzhydryl methyl ether*
3,3',4,5'-Tetrahydroxybibenzyl
(-)-Mesquitol*
5-Glucosyloxy-2-Hydroxybenzoic acid methyl ester
Methyl 3-O-glucosyl-4-hydroxybenzoate
5-Hydroxy-8-methoxycalamenene
4-(3,4,5-Trihydroxybenzoxy)benzoic acid
2,3,23-Trihydroxy-12-Oleanen-28-Oic Acid 3-O-[β-D-Glucopyranosyl-(1-4)-[α-L-Rhamnopyranosyl-(1-2)]]-α-L-Arabinopyranoside]
3-Chloroaniline
Hypoxanthine
Apiferol
Myricetin-3-O-galactoside*
4-Phenethylphenol*
(R)-3-Hydroxybutanoate
2-[(2R)-3-(3,4-dihydroxyphenyl)-2-hydroxypropyl]benzene-1,3,5-triol
5-Formylbenzofuran
Decursinol
(2R,3R)-3,5,7,3',5'-Pentahydroxyflavan
Malonate
Lycoclavanin
3-Hydroxybenzoate
Procyanidin B2
Isolariciresinol-9'-O-glucoside*
3,4-Methylenedioxycinnamaldehyde
12,13-Dehydrogeranylgeraniol
N-Hydroxypipecolic acid
Ent-14,15-Dinor-3-Cleroden-3-one
6-Hydroxyluteolin 7-rutinoside*
4-(2,6-Dihydroxybenzoyl)-3-Formyl-5-Hydroxybenzoic acid
13,28-Epoxy-11-ursen-3-one (Momordicinin)
Creatinine
trans-Cinnamate
D-Arabinono-1,4-lactone
Oleuropein
1-(4-Methoxyphenyl)-1-propanol
Melezitose
4-Aminobutanoate
8-(Beta-D-Glucopyranosyloxy)-2,7-Dimethyldec-2-enedioic acid
(R)-2,3-Dihydroxy-3-methylpentanoate
Uridine
Dihydrocoumarin
Cupresin B
LysoPC 20:2(2n isomer)
Hexadecanal
1-(β-D-Glucopyranosyl)-3-ethyl-4-methyl-1H-pyrrole-2,5-dione
9(S)-HPODE
4-Hydroxybenzoic acid glucoside*
Isobavachalcone glucoside
2-[4-(4-methylphenyl)-2-(propan-2-yl)oxan-4-yl]acetic acid
3-hydroxybenzoyl-β-D-glucose*
14,15-Dehydrocrepenynic acid
Nicotinamide
2-(Hydroxymethyl)Cyclohexa-2,5-Diene-1,4-Dione
Benzoylmalic acid
LysoPC 18:1
LysoPC 18:1(2n isomer)
9-Hydroxy-12-oxo-10(E),15(Z)-octadecadienoic acid
LPC(18:1/0:0)
Butyl isobutyl phthalate
9-Oxo-10,12-Octadecadienoic Acid
5-(6-aminopurin-9-yl)-2-ethyl-4-sulfanyloxolan-3-ol
Hexadecanedioate
5'-O-methyladenosine
Ethanolamine phosphate
9,10-Dihydroxystearate
5-methyl-2-[3,4,5-trihydroxy-6-(hydroxymethyl)oxan-2-yl]oxychromen-4-one
Methylthioadenosine sulfoxide
PI(18:1/0:0)
1-(4-Hydroxybenzoyl)Glucose;25545-07-7
Rubione D*
LysoPC 18:2
Zizybeoside II
Salidroside
Benzoylglycerol 2-glucuronide
3-O-(3,5-Dimethoxy-4-hydroxybenzoyl)-4-O-caffeoylquinic acid
8-(5-Hydroxy-2,6,6-Trimethylcyclohex-2-En-1-Yl)-6-Methyloct-5-En-2-one
(9S)-Hydroxyoctadecadienoic acid
2-[(1R,2R,4aS,8aS)-2-hydroxy-2,5,5,8a-tetramethyl-hexahydro-1H-naphthalen-1-yl]ethyl acetate
(9Z,11E)-(13S)-13-Hydroperoxyoctadeca-9,11-dienoic acid
D-Xylonate*
3-Hydroxyurs-5(6),12,18(19)-trien-28-oic acid (Uncargenin B)
LysoPC 12:0
Sphingosine
methyl alpha-D-fructofuranoside*
4-O-beta-D-Glucosyl-sinapate
LysoPE 18:2
1-(2,3-dihydroxypropoxy)-3-(((2-(dimethylamino)ethoxy)(hydroxy)phosphoryl)oxy)propan-2-yl (E)-hexadec-9-enoate
LysoPC 16:1*
Allatem
LysoPE 18:3
3'-O-Methylguanosine
2-Dehydro-3-deoxy-L-arabinonate
LysoPC 16:0
2-[4-[(1R,2R)-1,2-dihydroxypropyl]phenoxy]-6-(hydroxymethyl)oxane-3,4,5-triol
Glaziovine
Indole-3-lactic acid glucoside
D-Arabinonate*
1,5-Anhydro-D-glucitol
Decaethylene glycol
LysoPE 18:1(2n isomer)*
3-Hydroxy-5,7-Megastigmadien-9-one 3-O-[β-D-Glucopyranosyl-(1-6)-β-D-Glucopyranoside]
5-hydroxy-anthraquinone-2-carboxylic acid
[[5-(6-amino-7H-purin-7-yl)-3,4-dihydroxyoxolan-2-yl]methoxy]phosphonic acid*
L-Ornithine
LysoPE 18:1*
LysoPG 16:0
Phenylacetylglycerol 2-glucuronide
Isoxanthopterin
His-Trp
Syringin
Furanofructosyl-α-D-(6-mustard acyl)glucoside
Xanthurenic Acid 8-O-Glucoside
alpha-hexylcinnamaldehyde
Dicumarol
2-methoxycinnamaldehyde
LysoPC 18:3(2n isomer)
Teuhircoside
Pterohoonoid A
(2S)-1-O-palmitoleyl-3-O-beta-D-galactopyranosylglycerol
Phosphatidylethanolamine lyso 18:2*
Demethylsyringin
Zizybeoside I*
N-(1-Deoxy-1-fructosyl)Tryptophan
LysoPC 17:1
LysoPC 18:3
Benzoylglycerol 3-glucuronide
LysoPC 16:1(2n isomer)*
Elemol
1-Linoleoyl-2-Lysophosphatidic Acid Monobutylamine Ester*
1-(((2-(dimethylamino)ethoxy)(hydroxy)phosphoryl)oxy)-3-hydroxypropan-2-yl (9Z,12Z)-octadeca-9,12-dienoate*
Scutebarbolide C
5,19-Epoxycucurbita-6,23-diene-3,25-diol (Momordicoside I Aglycone)*
[(2S,3R,4S,5S,6R)-3,4,5-trihydroxy-6-(hydroxymethyl)oxan-2-yl](2E,4E)-5-[(1R,3S,5S,8S)-3,8-dihydroxy-1,5-dimethyl-6-oxabicyclo[3.2.1]octan-8-yl]-3-methylpenta-2,4-dienoate
LysoPE 18:2(2n isomer)*
D-Sorbitol
Hydroxy-Dihydrophaseic Acid glucoside
LysoPC 18:2(2n isomer)
LysoPC 16:2(2n isomer)*
8beta-d-Glucopyranosyloxy-4-methoxy-5-methyl-coumarin
Adenosine 2',3'-cyclic phosphate*
4-Hydroxy-3,5-dimethoxybenzyl alcohol
Panose*
LysoPE 14:0
UMP
Prolylproline
3',5'-Cyclic AMP
LysoPE 16:1*
LysoPC 16:2*
L-Glycyl-L-proline
3,19-Epoxy-3,22-dihydroxydammara-20,24-dien-26-oic acid δ-lactone (Semialactone)
CK0w-3
CK0w-2
CK0w-1
CK6w-1
CK6w-2

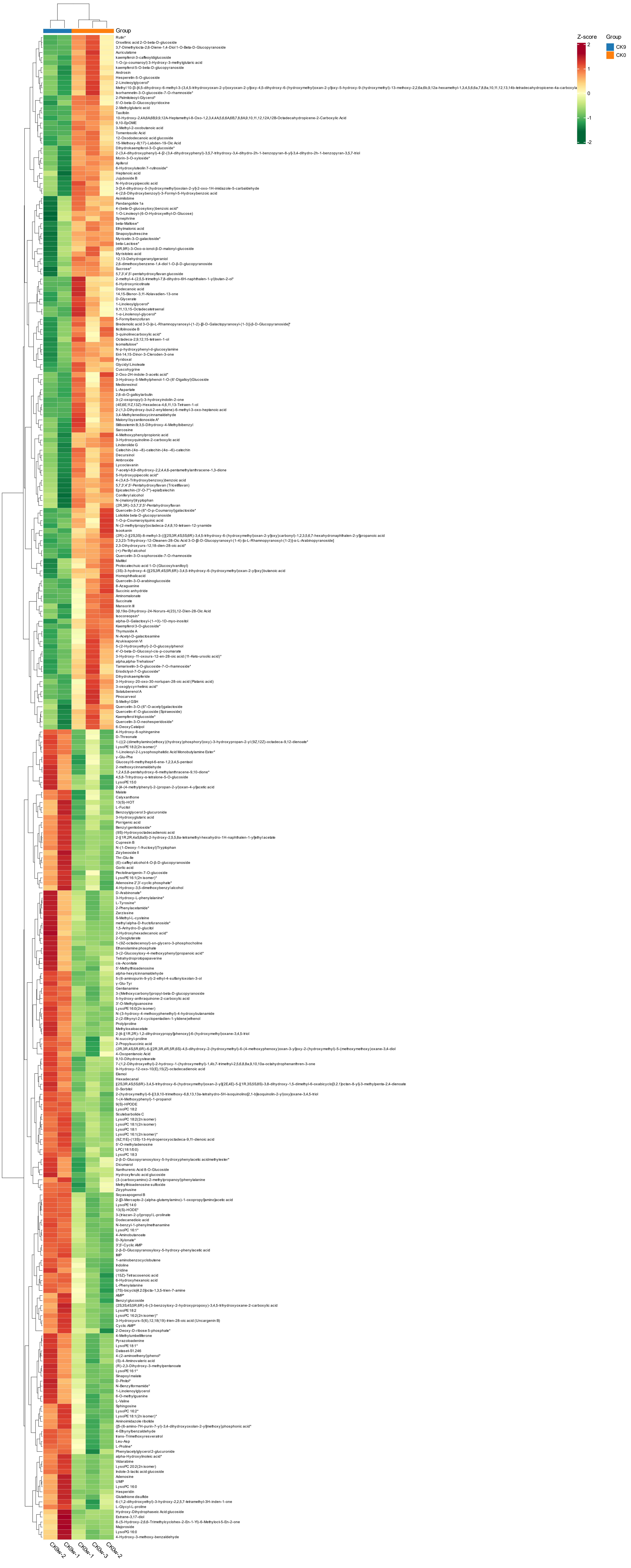
Group
Z-score
2
1
0
-1
-2
Group
CK9
CK0
CK9w-2
CK9w-1
CK0w-1
CK0w-3
CK0w-2

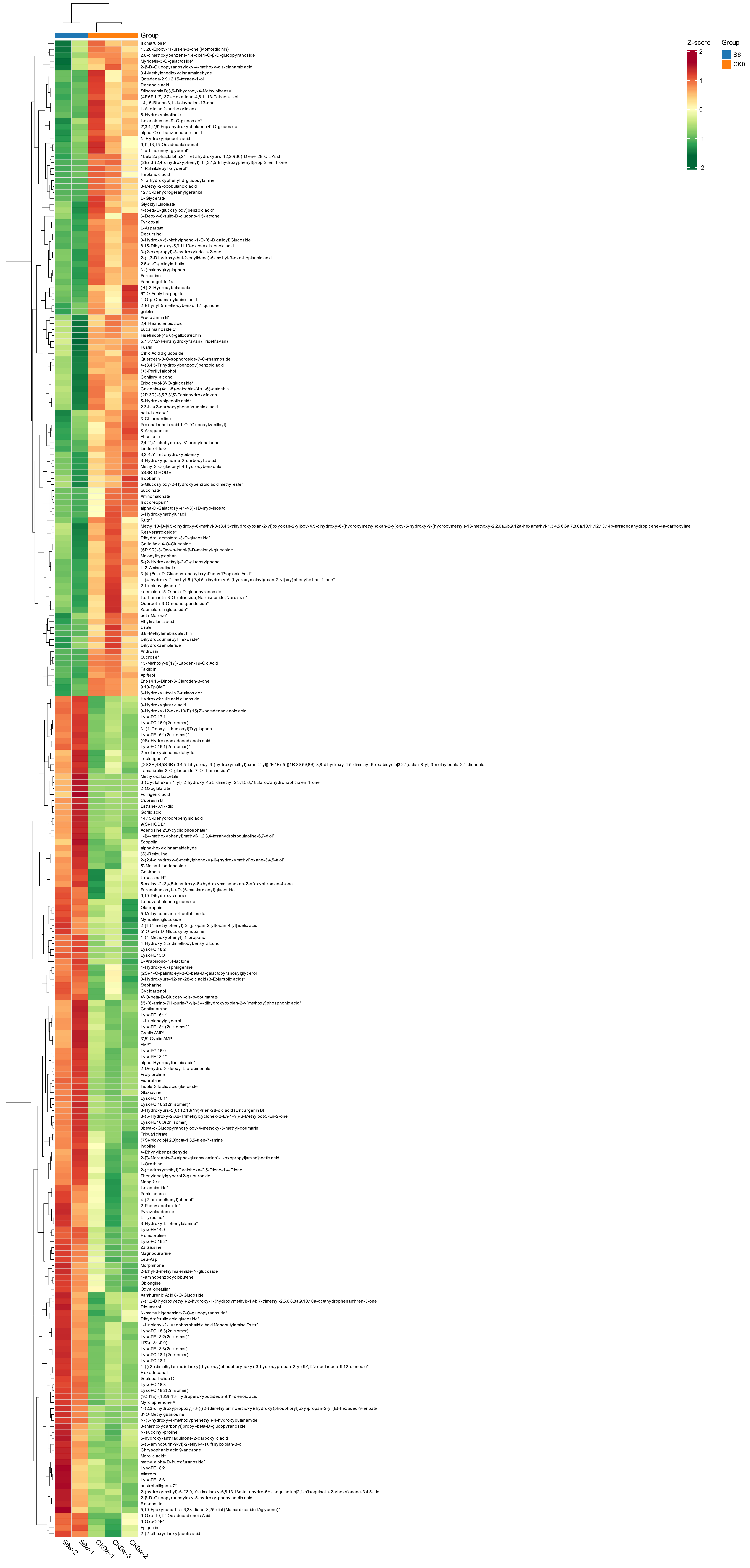

Group
Z-score
2
1
0
-1
-2
Group
S6
CK0
Isomaltulose*
13,28-Epoxy-11-ursen-3-one (Momordicinin)
2,6-dimethoxybenzene-1,4-diol 1-O-β-D-glucopyranoside
Myricetin-3-O-galactoside*
2-β-D-Glucopyranosyloxy-4-methoxy-cis-cinnamic acid
3,4-Methylenedioxycinnamaldehyde
Octadeca-2,9,12,15-tetraen-1-ol
Decanoic acid
Stilbostemin B;3,5-Dihydroxy-4-Methylbibenzyl
(4E,6E,11Z,13Z)-Hexadeca-4,6,11,13-Tetraen-1-ol
14,15-Bisnor-3,11-Kolavadien-13-one
L-Azetidine 2-carboxylic acid
6-Hydroxynicotinate
Isolariciresinol-9'-O-glucoside*
2',3,4,4',6'-Peptahydroxychalcone 4'-O-glucoside
alpha-Oxo-benzeneacetic acid
N-Hydroxypipecolic acid
9,11,13,15-Octadecatetraenal
1-α-Linolenoyl-glycerol*
1beta,2alpha,3alpha,24-Tetrahydroxyurs-12,20(30)-Diene-28-Oic Acid
(2E)-3-(2,4-dihydroxyphenyl)-1-(3,4,5-trihydroxyphenyl)prop-2-en-1-one
1-Palmitoleoyl-Glycerol*
Heptanoic acid
N-p-hydroxyphenyl-d-glucosylamine
3-Methyl-2-oxobutanoic acid
12,13-Dehydrogeranylgeraniol
D-Glycerate
Glycidyl Linoleate
4-(beta-D-glucosyloxy)benzoic acid*
6-Deoxy-6-sulfo-D-glucono-1,5-lactone
Pyridoxal
L-Aspartate
Decursinol
3-Hydroxy-5-Methylphenol-1-O-(6'-Digalloyl)Glucoside
8,15-Dihydroxy-5,9,11,13-eicosatetraenoic acid
3-(2-oxopropyl)-3-hydroxyindolin-2-one
2-(1,3-Dihydroxy-but-2-enylidene)-6-methyl-3-oxo-heptanoic acid
2,6-di-O-galloylarbutin
N-(malonyl)tryptophan
Sarcosine
Pandangolide 1a
(R)-3-Hydroxybutanoate
6''-O-Acetylharpagide
1-O-p-Coumaroylquinic acid
2-Ethynyl-5-methoxybenzo-1,4-quinone
grifolin
Arecatannin B1
2,4-Hexadienoic acid
Eucalmainoside C
Fisetinidol-(4α,6)-gallocatechin
5,7,3',4',5'-Pentahydroxyflavan (Tricetiflavan)
Fustin
Citric Acid diglucoside
Quercetin-3-O-sophoroside-7-O-rhamnoside
4-(3,4,5-Trihydroxybenzoxy)benzoic acid
(+)-Perillyl alcohol
Coniferyl alcohol
Eriodictyol-3'-O-glucoside*
Catechin-(4α→8)-catechin-(4α→6)-catechin
(2R,3R)-3,5,7,3',5'-Pentahydroxyflavan
5-Hydroxypipecolic acid*
2,3-bis(2-carboxyphenyl)succinic acid
beta-Lactose*
3-Chloroaniline
Protocatechuic acid 1-O-(Glucosylvanilloyl)
8-Azaguanine
Abscisate
2,4,2',4'-tetrahydroxy-3'-prenylchalcone
Linderolide G
3,3',4,5'-Tetrahydroxybibenzyl
3-Hydroxyquinoline-2-carboxylic acid
Methyl 3-O-glucosyl-4-hydroxybenzoate
5S,8R-DiHODE
Isookanin
5-Glucosyloxy-2-Hydroxybenzoic acid methylester
Succinate
Aminomalonate
Isocoreopsin*
alpha-D-Galactosyl-(1->3)-1D-myo-inositol
5-Hydroxymethyluracil
Rutin*
Methyl 10-[3-[4,5-dihydroxy-6-methyl-3-(3,4,5-trihydroxyoxan-2-yl)oxyoxan-2-yl]oxy-4,5-dihydroxy-6-(hydroxymethyl)oxan-2-yl]oxy-5-hydroxy-9-(hydroxymethyl)-13-methoxy-2,2,6a,6b,9,12a-hexamethyl-1,3,4,5,6,6a,7,8,8a,10,11,12,13,14b-tetradecahydropicene-4a-carboxylate
Resveratroloside*
Dihydrokaempferol-3-O-glucoside*
Gallic Acid 4-O-Glucoside
(6R,9R)-3-Oxo-α-ionol-β-D-malonyl-glucoside
Malonyltryptophan
5-(2-Hydroxyethyl)-2-O-glucosylphenol
L-2-Aminoadipate
3-[4-(Beta-D-Glucopyranosyloxy)Phenyl]Propionic Acid*
1-(4-hydroxy-2-methyl-6-[3,4,5-trihydroxy-6-(hydroxymethyl)oxan-2-yl]oxy}phenyl)ethan-1-one*
2-Linoleoylglycerol*
kaempferol 5-O-beta-D-glucopyranoside
Isorhamnetin-3-O-rutinoside;Narcissoside;Narcissin*
Quercetin-3-O-neohesperidoside*
Kaempferol triglucoside*
beta-Maltose*
Ethylmalonic acid
Urate
8,8'-Methylenebiscatechin
Dihydrocoumaroyl Hexoside*
Dihydrokaempferide
Androsin
Sucrose*
15-Methoxy-8(17)-Labden-19-Oic Acid
Taxifolin
Apiferol
Ent-14,15-Dinor-3-Cleroden-3-one
9,10-EpOME
6-Hydroxyluteolin 7-rutinoside*
Hydroxyferulic acid glucoside
3-Hydroxyglutaric acid
9-Hydroxy-12-oxo-10(E),15(Z)-octadecadienoic acid
LysoPC 17:1
LysoPC 16:0(2n isomer)
N-(1-Deoxy-1-fructosyl)Tryptophan
LysoPE 16:1(2n isomer)*
(9S)-Hydroxyoctadecadienoic acid
LysoPC 16:1(2n isomer)*
2-methoxycinnamaldehyde
Tectorigenin*
[(2S,3R,4S,5S,6R)-3,4,5-trihydroxy-6-(hydroxymethyl)oxan-2-yl](2E,4E)-5-[(1R,3S,5S,8S)-3,8-dihydroxy-1,5-dimethyl-6-oxabicyclo[3.2.1]octan-8-yl]-3-methylpenta-2,4-dienoate
Tamarixetin-3-O-glucoside-7-O-rhamnoside*
Methyloxaloacetate
3-(Cyclohexen-1-yl)-2-hydroxy-4a,5-dimethyl-2,3,4,5,6,7,8,8a-octahydronaphthalen-1-one
2-Oxoglutarate
Porrigenic acid
Cupresin B
Estrane-3,17-diol
Gorlic acid
14,15-Dehydrocrepenynic acid
9(S)-HODE*
Adenosine 2',3'-cyclic phosphate*
1-[(4-methoxyphenyl)methyl]-1,2,3,4-tetrahydroisoquinoline-6,7-diol*
Scopolin
alpha-hexylcinnamaldehyde
(S)-Reticuline
2-(2,4-dihydroxy-6-methylphenoxy)-6-(hydroxymethyl)oxane-3,4,5-triol*
5'-Methylthioadenosine
Gastrodin
Ursolic acid*
5-methyl-2-[3,4,5-trihydroxy-6-(hydroxymethyl)oxan-2-yl]oxychromen-4-one
Furanofructosyl-α-D-(6-mustard acyl)glucoside
9,10-Dihydroxystearate
Isobavachalcone glucoside
Oleuropein
5-Methylcoumarin-4-cellobioside
Myricetindiglucoside
2-[4-(4-methylphenyl)-2-(propan-2-yl)oxan-4-yl]acetic acid
5'-O-beta-D-Glucosylpyridoxine
1-(4-Methoxyphenyl)-1-propanol
4-Hydroxy-3,5-dimethoxybenzyl alcohol
LysoPC 18:2
LysoPE 15:0
D-Arabinono-1,4-lactone
4-Hydroxy-8-sphingenine
(2S)-1-O-palmitoleyl-3-O-beta-D-galactopyranosylglycerol
3-Hydroxyurs-12-en-28-oic acid (3-Epiursolic acid)*
Stepharine
Cycloartenol
4'-O-beta-D-Glucosyl-cis-p-coumarate
{[5-(6-amino-7H-purin-7-yl)-3,4-dihydroxyoxolan-2-yl]methoxy}phosphonic acid*
Gentianamine
LysoPE 16:1*
1-Linolenoylglycerol
LysoPE 18:1(2n isomer)*
Cyclic AMP*
3',5'-Cyclic AMP
AMP*
LysoPG 16:0
LysoPE 18:1*
alpha-Hydroxylinoleic acid*
2-Dehydro-3-deoxy-L-arabinonate
Prolylproline
Vidarabine
Indole-3-lactic acid glucoside
Glaziovine
LysoPC 16:1*
LysoPC 16:2(2n isomer)*
3-Hydroxyurs-5(6),12,18(19)-trien-28-oic acid (Uncargenin B)
8-(5-Hydroxy-2,6,6-Trimethylcyclohex-2-En-1-Yl)-6-Methyloct-5-En-2-one
LysoPE 16:0(2n isomer)
8beta-d-Glucopyranosyloxy-4-methoxy-5-methyl-coumarin
Tributylcitrate
(7S)-bicyclo[4.2.0]octa-1,3,5-trien-7-amine
Indoline
4-Ethynylbenzaldehyde
2-[[3-Mercapto-2-(alpha-glutamylamino)-1-oxopropyl]amino]acetic acid
L-Ornithine
2-(Hydroxymethyl)Cyclohexa-2,5-Diene-1,4-Dione
Phenylacetylglycerol 2-glucuronide
Mangiferin
Isotachioside*
Pantothenate
4-(2-aminoethenyl)phenol*
2-Phenylacetamide*
Pyrazoloadenine
L-Tyrosine*
3-Hydroxy-L-phenylalanine*
LysoPE 14:0
Homoproline
LysoPC 16:2*
Zarzissine
Magnocurarine
Leu-Asp
Morphinone
2-Ethyl-3-methylmaleimide-N-glucoside
1-aminobenzocyclobutene
Oblongine
Oxyallobetulin*
Xanthurenic Acid 8-O-Glucoside
7-(1,2-Dihydroxyethyl)-2-hydroxy-1-(hydroxymethyl)-1,4b,7-trimethyl-2,5,6,8,8a,9,10,10a-octahydrophenanthren-3-one
Dicumarol
N-methylhigenamine-7-O-glucopyranoside*
Dihydroferulic acid glucoside*
1-Linoleoyl-2-Lysophosphatidic Acid Monobutylamine Ester*
LysoPC 18:3(2n isomer)
LysoPE 18:2(2n isomer)*
LPC(18:1/0:0)
LysoPE 18:3(2n isomer)
LysoPC 18:1(2n isomer)
LysoPC 18:1
1-(((2-(dimethylamino)ethoxy)(hydroxy)phosphoryl)oxy)-3-hydroxypropan-2-yl (9Z,12Z)-octadeca-9,12-dienoate*
Hexadecanal
Scutebarbolide C
LysoPC 18:3
LysoPC 18:2(2n isomer)
(9Z,11E)-(13S)-13-Hydroperoxyoctadeca-9,11-dienoic acid
Myrciaphenone A
1-(2,3-dihydroxypropoxy)-3-(((2-(dimethylamino)ethoxy)(hydroxy)phosphoryl)oxy)propan-2-yl (E)-hexadec-9-enoate
3'-O-Methylguanosine
N-(3-hydroxy-4-methoxyphenethyl)-4-hydroxybutanamide
3-(Methoxycarbonyl)propyl-beta-D-glucopyranoside
N-succinyl-proline
5-hydroxy-anthraquinone-2-carboxylic acid
5-(6-aminopurin-9-yl)-2-ethyl-4-sulfanyloxolan-3-ol
Chrysophanic acid 9-anthrone
Morolic acid*
methyl alpha-D-fructofuranoside*
LysoPE 18:2
Aflatrem
LysoPE 18:3
austrobailignan-7*
2-(hydroxymethyl)-6-[[3,9,10-trimethoxy-6,8,13,13a-tetrahydro-5H-isoquinolino[2,1-b]isoquinolin-2-yl]oxy]oxane-3,4,5-triol
2-β-D-Glucopyranosyloxy-5-hydroxy-phenylacetic acid
Reseoside
5,19-Epoxycucurbita-6,23-diene-3,25-diol (Momordicoside I Aglycone)*
9-Oxo-10,12-Octadecadienoic Acid
9-OxoODE*
Epigoitrin
2-(2-ethoxyethoxy)acetic acid
S6w-2
S6w-1
CK0w-1
CK0w-3
CK0w-2

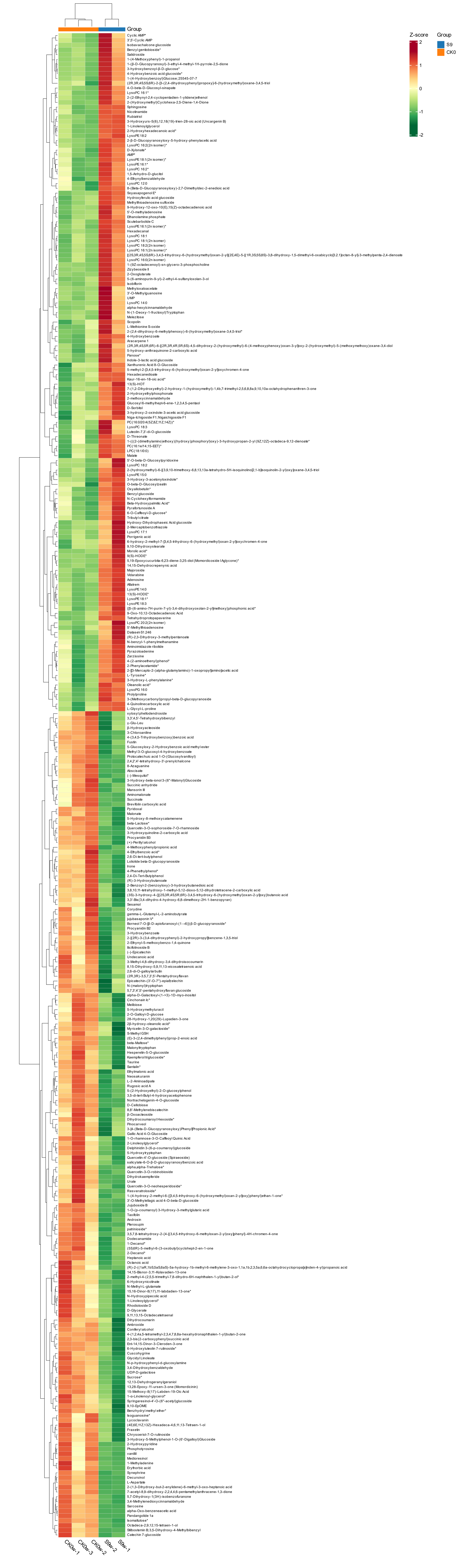

Group
Z-score
2
1
0
-1
-2
Group
S9
CK0
CK0w-1
CK0w-3
CK0w-2
S9w-2
S9w-1

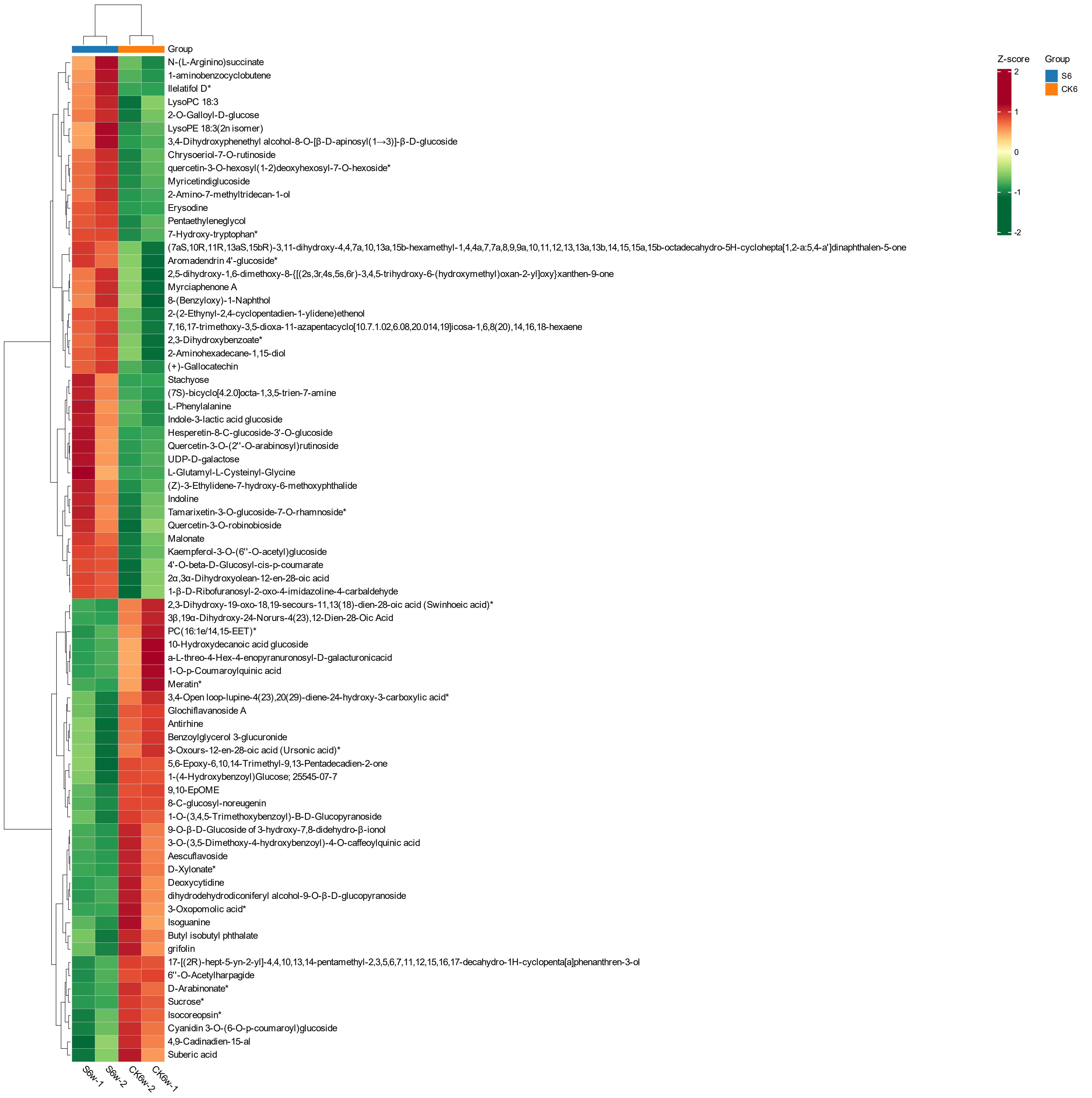

Group
N-(L-Arginino)succinate
1-aminobenzocyclobutene
Ilelatifol D*
LysoPC 18:3
2-O-Galloyl-D-glucose
LysoPE 18:3(2n isomer)
3,4-Dihydroxyphenethyl alcohol-8-O-[β-D-apinosyl(1→3)]-β-D-glucoside
Chrysoeriol-7-O-rutinoside
quercetin-3-O-hexosyl(1-2)deoxyhexosyl-7-O-hexoside*
Myricetindiglucoside
2-Amino-7-methyltridecan-1-ol
Erysodine
Pentaethyleneglycol
7-Hydroxy-tryptophan*
(7aS,10R,11R,13aS,15bR)-3,11-dihydroxy-4,4,7a,10,13a,15b-hexamethyl-1,4,4a,7,7a,8,9,9a,10,11,12,13,13a,13b,14,15,15a,15b-octadecahydro-5H-cyclohepta[1,2-a:5,4-a']dinaphthalen-5-one
Aromadendrin 4'-glucoside*
2,5-dihydroxy-1,6-dimethoxy-8-{[(2s,3r,4s,5s,6r)-3,4,5-trihydroxy-6-(hydroxymethyl)oxan-2-yl]oxy}xanthen-9-one
Myrciaphenone A
8-(Benzyloxy)-1-Naphthol
2-(2-Ethynyl-2,4-cyclopentadien-1-ylidene)ethenol
7,16,17-trimethoxy-3,5-dioxa-11-azapentacyclo[10.7.1.02,6.08,20.014,19]icosa-1,6,8(20),14,16,18-hexaene
2,3-Dihydroxybenzoate*
2-Aminohexadecane-1,15-diol
(+)-Gallocatechin
Stachyose
(7S)-bicyclo[4.2.0]octa-1,3,5-trien-7-amine
L-Phenylalanine
Indole-3-lactic acid glucoside
Hesperetin-8-C-glucoside-3'-O-glucoside
Quercetin-3-O-(2''-O-arabinosyl)rutinoside
UDP-D-galactose
L-Glutamyl-L-Cysteinyl-Glycine
(Z)-3-Ethylidene-7-hydroxy-6-methoxyphthalide
Indoline
Tamarixetin-3-O-glucoside-7-O-rhamnoside*
Quercetin-3-O-robinobioside
Malonate
Kaempferol-3-O-(6''-O-acetyl)glucoside
4'-O-beta-D-Glucosyl-cis-p-coumarate
2α,3α-Dihydroxyolean-12-en-28-oic acid
1-β-D-Ribofuranosyl-2-oxo-4-imidazoline-4-carbaldehyde
2,3-Dihydroxy-19-oxo-18,19-secours-11,13(18)-dien-28-oic acid (Swinhoeic acid)*
3β,19α-Dihydroxy-24-Norurs-4(23),12-Dien-28-Oic Acid
PC(16:1e/14,15-EET)*
10-Hydroxydecanoic acid glucoside
a-L-threo-4-Hex-4-enopyranuronosyl-D-galacturonicacid
1-O-p-Coumaroylquinic acid
Meratin*
3,4-Open loop-lupine-4(23),20(29)-diene-24-hydroxy-3-carboxylic acid*
Glochiflavanoside A
Antirhine
Benzoylglycerol 3-glucuronide
3-Oxours-12-en-28-oic acid (Ursonic acid)*
5,6-Epoxy-6,10,14-Trimethyl-9,13-Pentadecadien-2-one
1-(4-Hydroxybenzoyl)Glucose; 25545-07-7
9,10-EpOME
8-C-glucosyl-noreugenin
1-O-(3,4,5-Trimethoxybenzoyl)-B-D-Glucopyranoside
9-O-β-D-Glucoside of 3-hydroxy-7,8-didehydro-β-ionol
3-O-(3,5-Dimethoxy-4-hydroxybenzoyl)-4-O-caffeoylquinic acid
Aescuflavoside
D-Xylonate*
Deoxycytidine
dihydrodehydrodiconiferyl alcohol-9-O-β-D-glucopyranoside
3-Oxopomolic acid*
Isoguanine
Butyl isobutyl phthalate
grifolin
17-[(2R)-hept-5-yn-2-yl]-4,4,10,13,14-pentamethyl-2,3,5,6,7,11,12,15,16,17-decahydro-1H-cyclopenta[a]phenanthren-3-ol
6''-O-Acetylharpagide
D-Arabinonate*
Sucrose*
Isocoreopsin*
Cyanidin 3-O-(6-O-p-coumaroyl)glucoside
4,9-Cadinadien-15-al
Suberic acid
S6w-1
S6w-2
CK6w-2
CK6w-1
Z-score
2
1
0
-1
-2
Group
S6
CK6

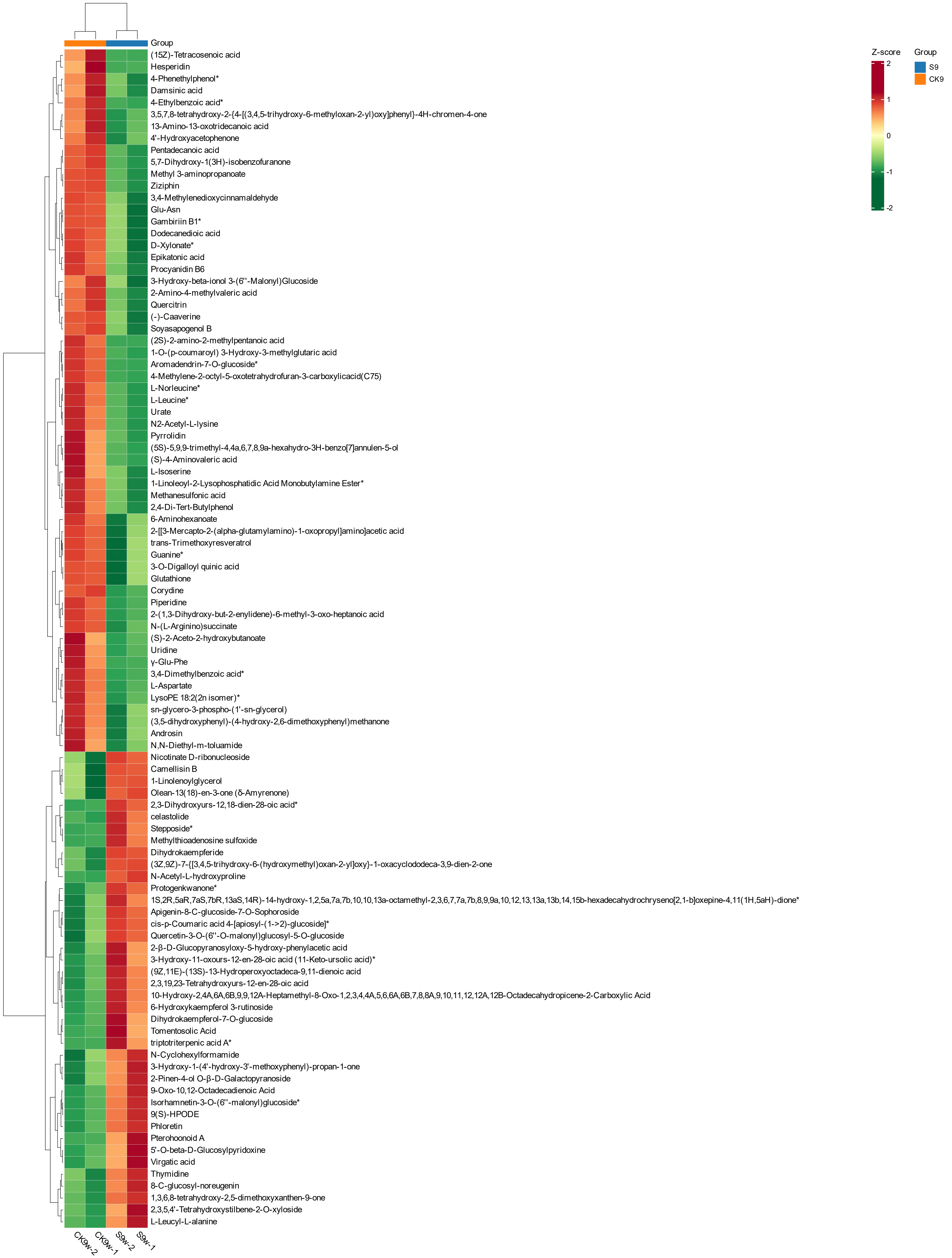
Group
Z-score
2
1
0
-1
-2
Group
S9
CK9
(15Z)-Tetracosenoic acid
Hesperidin
4-Phenethylphenol*
Damsinic acid
4-Ethylbenzoic acid*
3,5,7,8-tetrahydroxy-2-{4-[(3,4,5-trihydroxy-6-methyloxan-2-yl)oxy]phenyl}-4H-chromen-4-one
13-Amino-13-oxotridecanoic acid
4'-Hydroxyacetophenone
Pentadecanoic acid
5,7-Dihydroxy-1(3H)-isobenzofuranone
Methyl 3-aminopropanoate
Ziziphin
3,4-Methylenedioxycinnamaldehyde
Glu-Asn
Gambiriin B1*
Dodecanedioic acid
D-Xylonate*
Epikatonic acid
Procyanidin B6
3-Hydroxy-beta-ionol 3-(6''-Malonyl)Glucoside
2-Amino-4-methylvaleric acid
Quercitrin
(-)-Caaverine
Soyasapogenol B
(2S)-2-amino-2-methylpentanoic acid
1-O-(p-coumaroyl) 3-Hydroxy-3-methylglutaric acid
Aromadendrin-7-O-glucoside*
4-Methylene-2-octyl-5-oxotetrahydrofuran-3-carboxylicacid(C75)
L-Norleucine*
L-Leucine*
Urate
N2-Acetyl-L-lysine
Pyrrolidin
(5S)-5,9,9-trimethyl-4,4a,6,7,8,9a-hexahydro-3H-benzo[7]annulen-5-ol
(S)-4-Aminovaleric acid
L-Isoserine
1-Linoleoyl-2-Lysophosphatidic Acid Monobutylamine Ester*
Methanesulfonic acid
2,4-Di-Tert-Butylphenol
6-Aminohexanoate
2-[[3-Mercapto-2-(alpha-glutamylamino)-1-oxopropyl]amino]acetic acid
trans-Trimethoxyresveratrol
Guanine*
3-O-Digalloyl quinic acid
Glutathione
Corydine
Piperidine
2-(1,3-Dihydroxy-but-2-enylidene)-6-methyl-3-oxo-heptanoic acid
N-(L-Arginino)succinate
(S)-2-Aceto-2-hydroxybutanoate
Uridine
γ-Glu-Phe
3,4-Dimethylbenzoic acid*
L-Aspartate
LysoPE 18:2(2n isomer)*
sn-glycero-3-phospho-(1'-sn-glycerol)
(3,5-dihydroxyphenyl)-(4-hydroxy-2,6-dimethoxyphenyl)methanone
Androsin
N,N-Diethyl-m-toluamide
Nicotinate D-ribonucleoside
Camellisin B
1-Linolenoylglycerol
Olean-13(18)-en-3-one (δ-Amyrenone)
2,3-Dihydroxyurs-12,18-dien-28-oic acid*
celastolide
Stepposide*
Methylthioadenosine sulfoxide
Dihydrokaempferide
(3Z,9Z)-7-{[3,4,5-trihydroxy-6-(hydroxymethyl)oxan-2-yl]oxy}-1-oxacyclododeca-3,9-dien-2-one
N-Acetyl-L-hydroxyproline
Protogenkwanone*
1S,2R,5aR,7aS,7bR,13aS,14R)-14-hydroxy-1,2,5a,7a,7b,10,10,13a-octamethyl-2,3,6,7,7a,7b,8,9,9a,10,12,13,13a,13b,14,15b-hexadecahydrochryseno[2,1-b]oxepine-4,11(1H,5aH)-dione*
Apigenin-8-C-glucoside-7-O-Sophoroside
cis-p-Coumaric acid 4-[apiosyl-(1->2)-glucoside]*
Quercetin-3-O-(6''-O-malonyl)glucosyl-5-O-glucoside
2-β-D-Glucopyranosyloxy-5-hydroxy-phenylacetic acid
3-Hydroxy-11-oxours-12-en-28-oic acid (11-Keto-ursolic acid)*
(9Z,11E)-(13S)-13-Hydroperoxyoctadeca-9,11-dienoic acid
2,3,19,23-Tetrahydroxyurs-12-en-28-oic acid
10-Hydroxy-2,4A,6A,6B,9,9,12A-Heptamethyl-8-Oxo-1,2,3,4,4A,5,6,6A,6B,7,8,8A,9,10,11,12,12A,12B-Octadecahydropicene-2-Carboxylic Acid
6-Hydroxykaempferol 3-rutinoside
Dihydrokaempferol-7-O-glucoside
Tomentosolic Acid
triptotriterpenic acid A*
N-Cyclohexylformamide
3-Hydroxy-1-(4'-hydroxy-3'-methoxyphenyl)-propan-1-one
2-Pinen-4-ol O-β-D-Galactopyranoside
9-Oxo-10,12-Octadecadienoic Acid
Isorhamnetin-3-O-(6''-malonyl)glucoside*
9(S)-HPODE
Phloretin
Pterohoonoid A
5'-O-beta-D-Glucosylpyridoxine
Virgatic acid
Thymidine
8-C-glucosyl-noreugenin
1,3,6,8-tetrahydroxy-2,5-dimethoxyxanthen-9-one
2,3,5,4'-Tetrahydroxystilbene-2-O-xyloside
L-Leucyl-L-alanine
CK9w-2
CK9w-1
S9w-2
S9w-1

Table S1. Significant differential metabolites for different pairs of Dongzao cold storage treatments and storage weeks. If A vs. B is down-regulated, B has more metabolite than A; if up-regulated, A has more metabolite than B.

Group name	All sig diff	down regulated	up regulated
CK3_vs_CK0	145	45	100
CK6_vs_CK0	255	135	120
CK6_vs_CK3	137	75	62
CK6_vs_EA6	63	30	33
CK6_vs_S6	76	41	35
CK9_vs_CK0	299	138	161
CK9_vs_CK3	186	106	80
CK9_vs_CK6	109	50	59
EA3_vs_CK0	173	65	108
EA3_vs_CK0	173	65	108
EA3_vs_CK3	77	38	39
EA6_vs_CK0	271	142	129
EA6_vs_CK6	63	33	30
EA6_vs_EA3	168	85	83
EA9_vs_CK0	359	172	187
EA9_vs_CK9	89	51	38
EA9_vs_EA3	238	136	102
EA9_vs_EA6	139	73	66
M3_vs_CK0	203	66	137
M3_vs_CK3	64	33	31
M3_vs_S3	88	32	56
M6_vs_CK0	235	72	163
M6_vs_CK6	91	30	61
M6_vs_M3	133	63	70
M6_vs_M3	133	63	70
S3_vs_CK0	172	74	98
S3_vs_CK3	66	38	28
S3_vs_S6	132	71	61
S6_vs_CK0	251	110	141
S6_vs_M6	61	34	27
S6_vs_S3	132	61	71
S9_vs_CK0	315	173	142
S9_vs_CK9	99	59	40
S9_vs_EA9	144	72	72
S9_vs_S6	92	60	32
